# Serum proteomic profiling of sepsis patients reveals a protein-based diagnostic model, with metabolomic insights into carbapenem-resistant *Klebsiella pneumoniae* infection

**DOI:** 10.3389/fimmu.2026.1818068

**Published:** 2026-05-20

**Authors:** Juan He, Siqi Luo, Wenyun Xu, Yuanzhuo Chen, Guorong Liu, Jianguo Tang, Yong Yang, Bing Zhao, Li Ma, Huiqiu Sheng, Enqiang Mao

**Affiliations:** 1 Department of Pharmacy, Ruijin Hospital, Shanghai Jiao Tong University School of Medicine, Shanghai, China; 2 SpecAlly Life Technology Co., Ltd., Wuhan, China; 3 Department of Emergency, the Tenth People’s Hospital, Tongji University, Shanghai, China; 4 Department of Emergency, Gongli Hospital of Shanghai Pudong New Area, Shanghai, China; 5 Department of Emergency, the Fifth People’s Hospital of Shanghai, Fudan University, Shanghai, China; 6 Department of Emergency, Ruijin Hospital, Shanghai Jiao Tong University School of Medicine, Shanghai, China

**Keywords:** carbapenem-resistant Klebsiella pneumoniae, machine learning, metabolomics, proteomics, sepsis

## Abstract

**Introduction:**

Sepsis caused by carbapenem-resistant Klebsiella pneumoniae (CRKP) is associated with high mortality. Current research is predominantly pathogen-centric, creating a knowledge gap regarding the host’s systemic molecular response, which is critical for understanding outcomes and developing diagnostic strategies. This study aimed to characterize the host serum proteomic and metabolomic landscape of CRKP sepsis and to develop a proteomics-derived biomarker panel for differential diagnosis, while integrating metabolomic data to gain mechanistic insights into host pathways.

**Methods:**

Serum samples from sepsis patients, including culture-negative controls, and individuals infected with carbapenem-susceptible or carbapenem-resistant Klebsiella pneumoniae (CSKP and CRKP), underwent in-depth proteomic and metabolomic profiling. Differential expression, functional enrichment, and trend analyses were performed to characterize host molecular alterations associated with carbapenem resistance. Machine learning approaches were applied to construct diagnostic models based on host-derived molecular features. Candidate biomarker proteins were further validated using targeted parallel reaction monitoring (PRM) in an independent cohort.

**Results:**

We identified 85 differentially expressed proteins (DEPs) distinguishing CRKP from CSKP, enriched in viral infection-related pathways, antigen presentation, and phagosome function. Trend analysis revealed a protein cluster (51 proteins) with stepwise increased abundance from controls to CSKP to CRKP, implicating coagulation, immune signaling, and metabolic dysfunction. Based on proteomic data, a four-protein biomarker panel (IGHV1-8, ITGA2, PKP1, IGFBP6) effectively differentiated CRKP from CSKP (test AUC = 0.920). Targeted proteomic validation in an independent cohort confirmed the differential expression of key model proteins, including IGFBP6 (CRKP vs. CSKP) and APOA2 (CSKP vs. controls). Metabolomics identified 128 differential metabolites in CRKP vs. CSKP, with enrichment in thermogenesis and amino acid/fatty acid degradation pathways. Integrated pathway analysis of proteomic and metabolomic data highlighted dysregulation in cysteine/methionine metabolism and the folate-mediated one-carbon pool, with MAT2B as a key connecting protein.

**Conclusion:**

This first host-based multi-omics study of CRKP sepsis suggests distinct molecular signatures linked to resistance and disease severity. The independently validated biomarkers show high diagnostic potential, offering a preliminary foundation for early, non-invasive diagnosis and precision intervention strategies.

## Introduction

Sepsis is a life-threatening organ dysfunction caused by dysregulated host response to infection (1). As a leading global cause of death affecting millions annually with high mortality rates, sepsis poses persistent challenges to existing treatment strategies (2). Pneumonia serves as the most common origin of sepsis, and *Klebsiella pneumoniae* (KP) is a frequent pathogen in Gram-negative pneumonia and sepsis (3). In recent years, the emergence of carbapenem-resistant Klebsiella pneumoniae (CRKP) has posed particularly severe clinical challenges. CRKP infections are associated with extremely high morbidity and mortality (4–6), exhibiting a crude mortality rate span from 20 to 40%—significantly greater than that of carbapenem-susceptible Klebsiella pneumoniae (CSKP) infections—due to markedly limited therapeutic options. Although the poor prognosis of CRKP infection is widely recognized, the underlying host intrinsic molecular mechanisms responsible for worse clinical outcomes compared to susceptible strains remain poorly understood (7).

The current understanding of CRKP pathogenesis remains constrained by a pathogen-centric research paradigm. Most studies predominantly focus on the pathogen itself, employing *in vitro* cultures of KP for genomic, transcriptomic, proteomic and metabolomic analyses to investigate resistance mechanisms, virulence factors, and interactions with the gut microbiota (7–15). This pathogen-centric focus, however, has led to several critical knowledge gaps. First, integrated multi-omics analyses of systemic biological samples such as host blood remain largely unexplored. However, this pathogen-centric approach, while crucial for understanding bacterial resistance, fails to fully capture the complex, systemic pathophysiological responses and host immune alterations triggered by the infection within the host (16). The host’s response to infection, encompassing inflammatory cascades, immune cell functions, and metabolic reprogramming, is a critical determinant of sepsis outcome (17). Furthermore, while previous studies have identified individual proteins or metabolites associated with bacterial infections (10, 15), the integrated molecular landscape—spanning differential proteomic and metabolomic alterations across the spectrum from culture-negative sepsis to susceptible and resistant infection—remains poorly uncharacterized (13, 15). Finally, host-derived biomarkers capable of distinguishing CRKP infection and predicting prognosis are still lacking. Given that the initial recognition and management of sepsis largely occurs in the emergency department (ED), there is a pressing clinical need for host-derived biomarkers that could inform early, targeted antimicrobial decisions prior to the availability of culture and susceptibility results (18). A rapid, blood-based signature capable of distinguishing CRKP from CSKP infection could support more judicious empiric antibiotic selection in both the ED and intensive care unit (ICU), potentially reducing unnecessary broad−spectrum antibiotic exposure. Although predictive models based on clinical features and bacterial characteristics have been attempted (5, 7), biomarker panels based on host molecular signatures hold greater potential for early diagnosis and acute care management.

To address these knowledge gaps, we performed in-depth proteomic and metabolomic analyses of serum samples from patients with CRKP infection, CSKP infection, and sepsis controls with negative bacterial cultures. The specific aims of this study are: (1) to systematically delineate and compare the host serum molecular landscape (proteome and metabolome) of CRKP versus CSKP sepsis; (2) to identify key host molecular features and coordinated expression modules associated with carbapenem resistance; (3) to explore and validate a panel of host-derived biomarkers for distinguishing CRKP infection, and (4) to independently verify the abundance of crucial biomarker candidates using targeted mass spectrometry in a validation cohort. This work aims to provide a novel host-centered perspective on CRKP-associated mortality and establish a foundation for developing precision diagnostic and therapeutic strategies.

## Methods

### Study participants

The study participants were recruited from the emergency departments and intensive care units (ICUs) of Ruijin Hospital Affiliated with Shanghai Jiao Tong University School of Medicine, Shanghai Tenth People’s Hospital, Gongli Hospital of Shanghai Pudong New Area, and the Fifth People’s Hospital of Shanghai between April 1, 2025, and December 20, 2025. All enrolled patients were ≥18 years of age and were diagnosed with sepsis according to the Third International Consensus Definitions for Sepsis and Septic Shock (Sepsis-3) (1). The cohort was divided into a discovery set and an independent validation set, each containing 45 participants and stratified into three groups: sepsis patients with negative bacterial cultures (Con, n=15 per set), sepsis patients with confirmed CRKP infection (CRKP, n=15 per set), and sepsis patients with confirmed CSKP infection (CSKP, n=15 per set). The Con group comprised sepsis patients with negative results on all clinically indicated microbiological cultures. Inclusion criteria for the CRKP and CSKP groups included age ≥18 years, ICU stay ≥48 hours, and confirmed CRKP or CSKP infection, respectively. Exclusion criteria encompassed pre-existing CRKP/CSKP infection at admission, significant missing clinical data, and colonization without active infection.

### Serum collection and storage

Serum samples were collected at the time of sepsis diagnosis. Residual blood samples from routine clinical testing were used to minimize additional invasive procedures. Blood smears were prepared for microbiological confirmation of CRKP or CSKP. Serum was separated and aliquoted for storage at -80 °C to preserve sample integrity for subsequent multi-omics analyses.

### Covariates

A comprehensive set of clinical variables was collected for all participants, including demographic characteristics (sex, age, body mass index [BMI]) and laboratory parameters such as total bilirubin (TBIL), alanine aminotransferase (ALT), aspartate aminotransferase (AST), creatinine, blood urea nitrogen (BUN), procalcitonin (PCT), C-reactive protein (CRP), international normalized ratio (INR), neutrophil-to-lymphocyte ratio (NLR), red blood cell counts (RBC count), hemoglobin (Hb), red cell distribution width (RDW), platelet count (PLT count). Comorbidities including diabetes, heart disease, chronic obstructive pulmonary disease, liver disease, cerebrovascular disease, and kidney disease were also documented.

### Serum proteomics by LC-MS

Serum samples were processed using superparamagnetic iron oxide nanoparticle beads for protein enrichment. A 20 µL aliquot of each sample was diluted with loading buffer (10 mM Tris-Cl, 1 mM EDTA, 150 mM KCl, 0.05% CHAPS) and mixed with 1 mg of nanoparticle bead suspension, followed by incubation at 37 °C for 1 h. The beads were washed twice with loading buffer and once with CHAPS-free loading buffer (10 mM Tris-Cl, 1 mM EDTA, 150 mM KCl). After magnetic separation and supernatant removal, the protein-bound beads were subjected to reduction and alkylation using a lysis buffer (1% SDC, 100 mM Tris-HCl, pH 8.5, 10 mM TCEP, 40 mM CAA) at 60 °C for 30 min. The SDC concentration was then diluted below 0.5% by adding an equal volume of ddH_2_O, followed by digestion with 1 μg of trypsin at 37 °C overnight with agitation. The reaction was terminated by adding TFA, and the supernatant was desalted using SDB-RPS stage tips, vacuum-dried, and stored at −20 °C until LC-MS analysis (19).

LC-MS analysis was performed on an UltiMate 3000 RSLCnano system (Thermo) coupled to a timsTOF Pro mass spectrometer (Bruker) (20). Peptides were loaded onto a C18 trap column (75 μm × 2 cm, 3 μm particles, 100 Å, Thermo) and separated on an analytical column (75 μm × 15 cm, 1.7 μm particles, 100 Å, IonOpticks) using a gradient of mobile phase A (0.1% formic acid) and B (0.1% formic acid in acetonitrile) at a flow rate of 300 nL/min. Data were acquired in diaPASEF mode with the capillary voltage set to 1500 V (21). MS1 and MS2 scans covered a range of 100–1700 m/z, with an ion mobility range of 0.6–1.6 Vs/cm². Accumulation and ramp times were set to 50 ms. diaPASEF windows were defined using timsControl based on m/z–ion mobility distributions. Collision energy decreased linearly from 59 eV at 1/K_0_ = 1.6 Vs/cm² to 20 eV at 1/K_0_ = 0.6 Vs/cm².

Raw DIA data were processed using DIA-NN (version 1.9.2) in library-free mode. Spectra were searched against the HUMAN UniProt database (release 20250113) (22) with the following parameters: Precursor ion generation enabled; Trypsin/P specificity with up to 2 missed cleavages; Carbamidomethyl (C) as fixed modification; Oxidation (M) and Acetylation (protein N-term) as variable modifications; MS1 and MS2 mass tolerances set to 15 ppm; Match-between-runs (MBR) and heuristic protein inference were enabled; FDR threshold set to 1%. Protein quantification was performed using the MaxLFQ algorithm for intensity normalization (23).

### Targeted proteomic validation by parallel reaction monitoring

To independently validate the abundance of all proteins included in the discovery-phase diagnostic models, a targeted PRM assay was performed on the separate validation cohort (n=45; 15 Con, 15 CSKP, 15 CRKP). The samples were enriched using superparamagnetic iron oxide nanoparticles. Twenty μL of the sample was diluted with loading buffer (10 mM Tris-Cl, 1 mM EDTA, 150 mM KCl, 0.05% CHAPS) and mixed with 1 mg of magnetic beads. The mixture was incubated at 37 °C for 1 h. The magnetic beads were washed twice with the loading buffer, followed by one wash with a CHAPS-free buffer (10 mM Tris-Cl, 1 mM EDTA, 150 mM KCl). The magnetic beads were captured on a magnetic rack, and the supernatant was discarded to obtain the protein-enriched magnetic beads. The sample was then added with lysis buffer (1% SDC/100 mM Tris-HCl, pH=8.5/10 mM TCEP/40 mM CAA) and incubated at 60 °C for 30 minutes for protein reduction and alkylation. An equal volume of ddH2O was added to dilute the SDC to a concentration below 0.5%, and 1 μg of trypsin was added. The mixture was incubated overnight at 37 °C for enzymatic digestion. The next day, TFA was used to bring the pH down to 6.0 to end the digestion. After centrifugation, the supernatant was subjected to peptide purification using self-made SDB-RPS desalting columns. The peptide eluate was vacuum dried and stored at -20 °C for later use.

Combined DIA (timsTOF Pro) and PRM (Q Exactive HF) acquisition for spectral library construction and targeted quantification. For spectral library construction, DIA was performed on a timsTOF Pro mass spectrometer (Bruker Daltonics) coupled online with an UltiMate 3000 RSLCnano system (Thermo Fisher Scientific) via a CaptiveSpray nano ion source (Bruker Daltonics). Peptide samples were injected into a C18 Trap column (75 µm*2 cm, 3 µm particle size, 100 Å pore size, Thermo), and separated on a reversed-phase C18 analytical column (75 µm*15 cm, 1.7 µm particle size, 100 Å pore size, IonOpticks). Mobile phase A (0.1% formic acid in water) and mobile phase B (0.1% formic acid in ACN) were used to establish the separation gradient at a flow rate of 300 nL/min. The MS was operated in PASEF mode. The capillary voltage was set to 1500 V. The MS and MS/MS spectra were acquired from 100 to 1700 m/z. The ion mobility was scanned from 0.6 to 1.6 Vs/cm^2^. The acquisition cycle comprised one full MS scan and ten PASEF MS/MS scans. Singly charged precursors were filtered out by ion mobility. The collision energy was ramped linearly as a function of the mobility from 59 eV at 1/K_0_ = 1.6 Vs/cm^2^ to 20 eV at 1/K_0_ = 0.6 Vs/cm^2^.

For targeted protein quantification, parallel reaction monitoring (PRM) was carried out on a Q Exactive HF mass spectrometer (Thermo Fisher Scientific) coupled to an UltiMate 3000 RSLCnano system via a Nanospray Flex ion source (Thermo). Peptide separation was performed on a C18 trap column (75 µm*2 cm, 3 µm particle size, 100 Å pore size, Thermo) and a homemade reversed−phase C18 analytical column (75 µm × 25 cm, 1.9 µm, 100 Å, ReproSil−Pur C18−AQ resin) at 300 nL/min using mobile phase A (0.1% formic acid/3% DMSO/97% H_2_O) and mobile phase B (0.1% formic acid/3% DMSO/97% acetonitrile). PRM scans consisted of one full MS event followed by PRM MS/MS events in a cycle. AGC Target value for the full MS scan was 3E6 charges in the 300-1500 m/z range with a maximum injection time of 30 ms and a resolution of 60,000 at m/z 200. Target ions were submitted to MS/MS in the HCD cell. PRM MS/MS scans were acquired at a resolution of 15,000 at m/z 200 with an AGC target value of 2E5, a maximum injection time of 20 ms. Target precursor ions were fragmented by higher−energy collisional dissociation (HCD) using a normalized collision energy of 27.

DDA-MS raw data were analyzed using MaxQuant with the Andromeda database search algorithm and the FragPipe platform equipped with MSFragger algorithm (24). Spectra files were searched against the self-defined protein sequence database downloaded from UniProt to generate spectral library from search results (22). The search results served as the basis for establishing a spectral library in the Skyline software. The search results were imported into the Skyline software to build library. The peptide setting parameters of Skyline were as follows: the enzyme was set as Trypsin [KR/P], and maximum missed cleavages were set to 0. The peptide length was set as 7–25, and the cysteine alkylation was set as fixed modification. The transition setting parameters of Skyline are: precursor charges were set as 2, 3, and 4, ion charges were set as 1, 2, and 3, and ion types were set as b, y, and p. The product ions were set from ion 3 to the last ion, the ion match tolerance was set as 0.5 m/z. To determine the relative abundance of the target peptides, we used the fragment area and its background area to calculate the relative abundance of the target peptides. The PRM isolation list is provided in **Supplementary Table S1**.

### Serum metabolomics by LC-MS

Serum samples were thawed on ice and vortexed for 10 s. A 50 μL aliquot was mixed with 300 μL of extraction solution (acetonitrile:methanol = 1:4, v/v) containing internal standards. After vortexing for 3 min, the mixture was centrifuged at 12,000 rpm for 10 min at 4 °C. Then, 200 μL of supernatant was transferred to a new tube, maintained at −20 °C for 30 min, and centrifuged again at 12,000 rpm for 3 min at 4 °C. Finally, 180 μL of supernatant was collected for LC-MS analysis.

The sample extracts were analyzed using an LC-ESI-MS/MS system (UPLC, ExionLC AD, https://sciex.com.cn/; MS, QTRAP^®^ System, https://sciex.com/). Chromatographic separation was performed on a Waters ACQUITY UPLC HSS T3 C18 column (1.8 μm, 2.1 × 100 mm) using an ExionLC AD system (Sciex). The mobile phase consisted of (A) ultrapure water with 0.1% formic acid and (B) acetonitrile with 0.1% formic acid. The gradient elution was as follows: 0 min, 95:5 (A:B, v/v); 2.0 min, 80:20; 5.0 min, 40:60; 6.0 min, 1:99; 7.5 min, 1:99; 7.6 min, 95:5; and 10.0 min, 95:5. The flow rate was 0.4 mL/min, column temperature was maintained at 40 °C, and the injection volume was 2 μL.

Mass spectrometric detection was conducted on a QTRAP^®^ instrument (Sciex) equipped with an electrospray ionization (ESI) source. The ESI parameters were set as follows: source temperature 500 °C; ion spray voltage 5500 V (positive) and −4500 V (negative); ion source gas I, gas II, and curtain gas at 55, 60 and 25 psi, respectively; collision gas set to high. Instrument calibration was performed using 10 and 100 μmol/L polypropylene glycol solutions. Specific MRM transitions were monitored for each metabolite according to their elution period (25).

### Data processing and statistical analysis

For proteomic data, contaminant proteins were removed, and proteins with missing values in more than 70% of samples were filtered out. The remaining data were log-transformed, and missing values were imputed using a down-shifted normal distribution (mean = -1.8 × SD, width = 0.25 × SD) to simulate intensities near the detection limit. Proteins missing in more than 50% of samples in both comparison groups were excluded. Differentially expressed proteins (DEPs) were identified using an equal-variance t-test with |log_2_FC| ≥0.58 and p <0.05, excluding proteins supported by only one peptide.

For metabolomic data, missing values were filled with one-fifth of the minimum value per metabolite. Raw peak areas were log_2_−transformed. For each metabolite, data were then mean−centred and scaled by Z−score. Quality control was performed by calculating the coefficient of variation (CV) in QC samples, and metabolites with CV <0.3 were retained. No additional normalisation was applied, as all samples were processed in a single batch without systematic batch effects. Differentially expressed metabolites (DEMs) were identified based on variable importance in projection (VIP) values from orthogonal partial least squares discriminant analysis (OPLS-DA) models and p-values from statistical tests. For two-group comparisons, metabolites with VIP >1 and p <0.05 (Student’s t-test) were selected, while for multi-group comparisons, VIP >1 and p <0.05 (ANOVA) were applied. Prior to OPLS-DA, data were log_2_-transformed and mean-centered. Permutation testing (200 permutations) was performed to validate model robustness (26, 27).

Clinical variables were summarized as mean ± SD for normally distributed continuous variables, median with interquartile range for non-normal variables, and percentages for categorical variables. Group comparisons were performed using Student’s t-test (normal continuous), Wilcoxon rank-sum test (non-normal continuous), chi-square or Fisher’s exact test (categorical), and Wilcoxon signed-rank test for ordinal variables.

### Bioinformatics analysis

Based on the union of DEPs, we performed Mfuzz-based trend clustering to group proteins with similar expression patterns across disease progression. Each cluster’s expression profile was visualized by plotting the mean abundance levels, and proteins within each cluster were subjected to Gene Ontology (GO) and Kyoto Encyclopedia of Genes and Genomes (KEGG) enrichment analyses. One biologically relevant cluster was selected for further investigation. To analyze metabolite patterns, Z-score-normalized relative abundances of all differential metabolites identified across the comparison groups were subjected to K-means clustering using R (base package). The most relevant peptides from the selected cluster were identified after setting membership of cluster ≥0.4, relative standard deviation (RSD) ≤0.4, and a p-value <0.05 among samples. Box plots were used to visualize the expression levels of key proteins across all groups, and Pearson correlation analysis was conducted to evaluate the associations between clinical parameters and key protein expression.

Functional annotation and enrichment analysis were performed using the following databases: GO (http://www.geneontology.org/) for biological process (BP), cellular component (CC), and molecular function (MF) (28); KEGG (https://www.kegg.jp/kegg/) for pathway analysis (29); and Disease Ontology (DO, https://disease-ontology.org/) for disease association (30). Terms with a hypergeometric test p-value <0.05 were considered significantly enriched. Protein–protein interaction (PPI) data were obtained from the STRING database (https://string-db.org) using a combined interaction score threshold greater than 0.4 (31). The resulting network was optimized using a degree-based layout to identify hub proteins. KEGG pathway integrative analysis was performed using MetaboAnalyst 6.0 (https://www.metaboanalyst.ca/) (32).

Key proteins annotated as immune related were identified using the ImmPort database (https://www.immport.org/home) (33) and further analyzed by KEGG pathway enrichment (p < 0.05). Tissue specificity of key proteins was assessed using the Human Protein Atlas (HPA, https://www.proteinatlas.org/), and highly tissue-enriched proteins were highlighted (34). Drug–protein interactions were queried using the Drug–Gene Interaction Database (DGIdb, https://dgidb.org) (35). Drugs targeting single or multiple key proteins were identified, and the top five multi-target drugs were selected for visualization. Additional databases—AnimalTFDB4, PhosphoSitePlus, TCDB, TTD, and CellChatDB—were used to annotate transcription factors, kinases, ion channels, disease targets, and receptor proteins among the key proteins, and drug–protein interaction networks were constructed.

### Machine learning for biomarker panel development

Proteins supported by only one peptide were excluded to improve protein confidence. The dataset was randomly split into training (70%) and test (30%) sets. In the training set, LASSO regression with cross-validation was used to select candidate proteins. For each candidate protein, we (1) plotted ROC curves and calculated the area under the curve (AUC) to evaluate diagnostic performance; (2) performed univariate logistic regression to assess the association with clinical outcomes; and (3) conducted Pearson correlation analysis with continuous clinical variables.

To identify the optimal algorithm for constructing a protein biomarker panel, we compared three commonly used algorithms: logistic regression, random forest, and XGBoost (36, 37). All possible combinations of candidate proteins were used to build models in the training set. Models were evaluated based on the AUC, accuracy, and Brier score. The final model was selected based on the highest test AUC (with a training AUC < 1 to avoid overfitting), and accuracy was used as a tiebreaker. Model performance was assessed in both training and test sets using ROC curves (for discrimination), Hosmer–Lemeshow tests and calibration curves (for calibration), and decision curve analysis (DCA, for clinical utility).

All statistical and bioinformatic analyses were performed using R software (version 4.5.1, Vienna, Austria) and Python version 3.10.12. The following R packages were used for specific analyses: MetaboAnalystR for metabolomics analysis (38); pROC (v1.18.5) and ROCit (v2.1.2) for ROC curve analysis; rms (v8.0.0) for DCA, calibration curves, and nomograms; ResourceSelection (v0.3.6) for Hosmer–Lemeshow tests; corrplot (v0.95) for Pearson correlation visualization; caret (v7.0.1) for data splitting; glmnet (v4.1.9) for LASSO regression; Mfuzz (v2.68.0) for trend clustering; and stats and rms for univariate and multivariable logistic regression. Machine learning model construction and interpretation were implemented using the sklearn (v1.7.0), statsmodels (v0.14.4), and SHAP (v0.46.0) libraries in Python. A two-sided p value < 0.05 was considered statistically significant.

## Results

### Patient characteristics

Our study cohort comprised a discovery set of 45 participants, including 15 with culture-negative (Con) sepsis, 15 with CSKP sepsis, and 15 with CRKP sepsis. An independent validation set of 45 participants with identical group distribution was also included. Serum samples were collected from all individuals for subsequent proteomic analysis. Clinical characteristics of the discovery cohort are summarized in **Table 1**. The discovery cohort was characterized by a median age of 50 (IQR: 36–64) years, with 31 men (68.89%) and a high prevalence of cerebrovascular disease (21 individuals; 46.67%). The age, sex, and BMI of the Con, CSKP, and CRKP were similar among these groups. Significant differences were observed among the three groups in NLR, PCT, CRP, RBC count, and Hb levels (all P < 0.001). In the CSKP and CRKP groups, the levels of NLR, PCT, and CRP were significantly elevated above the normal reference range, while RBC counts and Hb levels fell markedly below the lower limit of normal. These abnormalities were more pronounced in the CRKP group than in the CSKP group, indicating that the CRKP group exhibited the most severe inflammatory response and infection-related consumptive pathology. Baseline characteristics in the validation cohort were similar to those in the discovery cohort, with the CRKP group showing significant inflammation (**Supplementary Table S2**).

**Table 1:** Descriptive characteristics of discovery cohort.

Characteristic	Total N = 45	Con n = 15	CSKP n = 15	CRKP n = 15	p-value
Age (yrs, median [IQR])	50.0 [36.0, 64.0]	40.0 [34.5, 56.0]	55.0 [38.0, 64.5]	53.0 [38.0, 67.0]	0.305
Sex					1.000
Male	31 (68.89)	10 (66.67)	11 (73.33)	10 (66.67)	
Female	14 (31.11)	5 (33.33)	4 (26.67)	5 (33.33)	
BMI (kg/m^2^, mean ± SD)	22.85 ± 4.46	24.79 ± 5.42	21.03 ± 2.99	22.73 ± 4.09	0.066
TBIL (μmol/L, median [IQR])	16.00 [11.80, 27.00]	15.50 [10.30, 17.70]	16.40 [12.90, 46.20]	18.80 [15.05, 27.00]	0.180
ALT (U/L, median [IQR])	26.00 [14.00, 46.00]	22.00 [13.00, 41.50]	26.00 [13.50, 69.50]	44.00 [15.50, 45.50]	0.815
AST (U/L, median [IQR])	34.00 [18.00, 51.00]	23.00 [17.50, 43.00]	40.00 [19.00, 57.50]	38.00 [20.00, 46.00]	0.635
Creatinine (μmol/L, median [IQR])	68.00 [58.00, 108.00]	68.00 [64.00, 78.50]	75.00 [59.50, 105.50]	66.00 [44.50, 125.50]	0.952
BUN (mmol/L, median [IQR])	5.30 [4.40, 11.70]	4.50 [3.35, 5.95]	4.90 [4.40, 11.40]	8.30 [4.70, 17.30]	0.040
NLR (median [IQR])	6.52 [2.46, 17.36]	2.25 [1.57, 2.62]	10.00 [6.52, 17.14]	17.58 [8.64, 38.56]	< 0.001
PCT (ng/mL, median [IQR])	0.27 [0.07, 2.75]	0.07 [0.04, 0.18]	0.72 [0.17, 2.22]	2.33 [0.44, 11.92]	< 0.001
CRP (mg/L, median [IQR])	40.16 [5.00, 105.00]	5.00 [3.00, 9.50]	40.16 [8.90, 125.20]	105.00 [83.00, 122.60]	< 0.001
WBC count (median [IQR])	6.59 [4.51, 9.00]	5.56 [4.85, 6.31]	8.20 [0.11, 10.09]	8.47 [5.73, 13.46]	0.099
RBC count (mean ± SD)	3.23 ± 1.03	4.19 ± 0.69	2.83 ± 1.02	2.68 ± 0.56	< 0.001
Hb (mean ± SD)	97.18 ± 31.89	125.47 ± 23.93	87.27 ± 30.93	78.80 ± 18.49	< 0.001
RDW (median [IQR])	13.50 [12.90, 15.00]	13.30 [12.30, 13.95]	13.20 [13.00, 14.60]	14.50 [13.15, 15.55]	0.217
PLT (mean ± SD)	201.67 ± 135.09	268.60 (103.49)	148.53 (147.49)	187.87 (129.85)	0.042
INR (median [IQR])	1.09 [1.01, 1.21]	1.02 [0.97, 1.08]	1.09 [1.00, 1.18]	1.21 [1.16, 1.29]	0.002
Diabetes (n, %)					0.049
0	29 (64.44)	6 (40.00)	11 (73.33)	12 (80.00)	
1	16 (35.56)	9 (60.00)	4 (26.67)	3 (20.00)	
Heart disease (n, %)					1.000
0	38 (84.44)	13 (86.67)	12 (80.00)	13 (86.67)	
1	7 (15.56)	2 (13.33)	3 (20.00)	2 (13.33)	
COPD (n, %)					1.000
0	41 (91.11)	14 (93.33)	13 (86.67)	14 (93.33)	
1	4 (8.89)	1 (6.67)	2 (13.33)	1 (6.67)	
Liver disease (n, %)					0.174
0	31 (68.89)	10 (66.67)	8 (53.33)	13 (86.67)	
1	14 (31.11)	5 (33.33)	7 (46.67)	2 (13.33)	
Cerebrovascular disease (n, %)					0.090
0	24 (53.33)	5 (33.33)	11 (73.33)	8 (53.33)	
1	21 (46.67)	10 (66.67)	4 (26.67)	7 (46.67)	
Kidney disease (n, %)					1.000
0	40 (88.89)	14 (93.33)	13 (86.67)	13 (86.67)	
1	5 (11.11)	1 (6.67)	2 (13.33)	2 (13.33)	

Con, culture-negative controls; CSKP, carbapenem-susceptible Klebsiella pneumoniae; CRKP, carbapenem-resistant Klebsiella pneumoniae; BMI, body mass index; TBIL, total bilirubin; ALT, Alanine aminotransferase; AST, Aspartate aminotransferase; Creatinine; BUN, blood urea nitrogen; NLR, neutrophil-to-lymphocyte ratio; PCT, procalcitonin; CRP, C-reactive protein; WBC count, white blood cell count; RBC count, red blood cell count; Hb, hemoglobin; RDW, red cell distribution width; PLT, platelet count; INR, international normalized ratio; COPD, chronic obstructive pulmonary disease.

### Serum proteomic profiling and differential protein analysis

**Figure 1:**
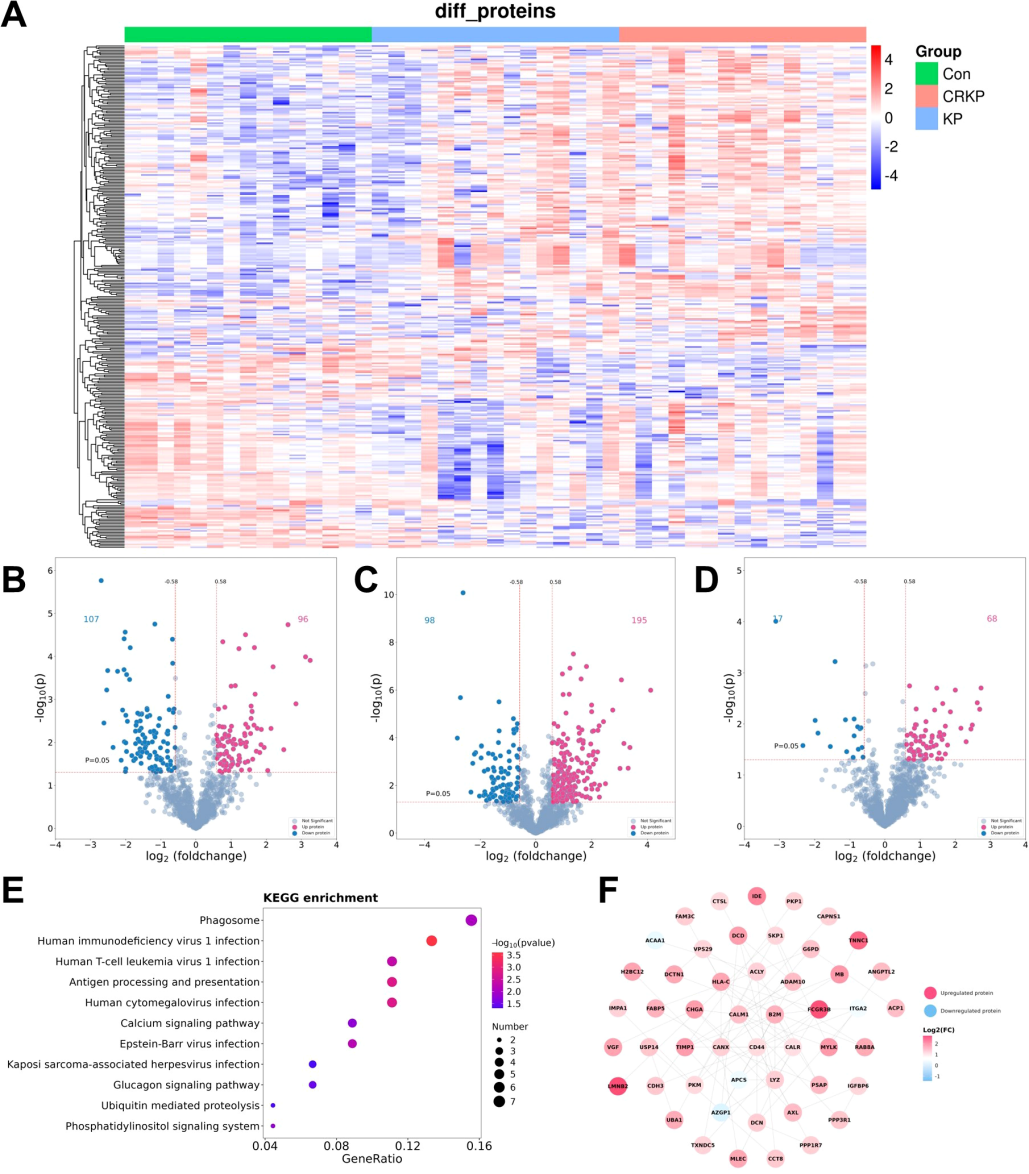
Differential analysis in proteins expression levels. **(A)** Heatmap of differential expressed proteins (DEPs) from the proteomics data. Red: upregulated proteins; blue: downregulated proteins. **(B–D)** Volcano plots of DEPs from the proteomics data for each pairwise comparison (|log2FC| ≥0.58 (vertical dashed line) and P <0.05 (horizontal dashed line). Gray: proteins that were not significantly deregulated; red: upregulated proteins; blue: downregulated proteins. **(E)** KEGG-based enrichment analysis of DEPs between the KP group and the Con group (two-sided hypergeometric test; P ≤0.05). KEGG terms were sorted by P-value, and top 15 terms were displayed. **(F)** Protein-protein interaction (PPI) network analysis diagram of differential proteins between the CRKP group and the KP group. Differential proteins are represented as circles, and the color of each protein varies according to the log2FC value.

We acquired proteome profiles of all participants using a data-independent acquisition (DIA) strategy. Comprehensive quality control metrics for proteomic data acquisition and sample reproducibility are provided in **Supplementary Figure S1** and **Supplementary Methods**. Proteins with a missing data proportion of greater than 70% across all samples, as well as those with a missing data proportion exceeding 50% in both groups, were removed. The hierarchical clustering heatmap showed a total of 339 DEPs, with significant differences in the protein expression patterns of each group (**Figure 1A**). A total of 203 and 293 DEPs were identified when comparing the KP group (CSKP and CRKP) to the Con group, respectively (**Figures 1B, C**). Notably, 85 DEPs were detected between CRKP and CSKP (**Figure 1D**), with the DEPs being closely associated with infection pathways (e.g., Human immunodeficiency virus 1 and Human cytomegalovirus infection), immune processes such as antigen processing, and presentation and phagosome, as well as fundamental cellular signaling pathways including the phosphatidylinositol signaling system and calcium signaling pathway (**Figure 1E**). Protein-protein interaction (PPI) network analysis of the 85 DEPs via the STRING database (confidence score >0.4) revealed an interconnected network comprising 74 nodes and 81 edges (**Figure 1F**), with CD44 and B2M identified as the top hub proteins based on degree centrality (degree = 11).

### Protein biomarkers for diagnosing CSKP and CRKP patients

**Figure 2:**
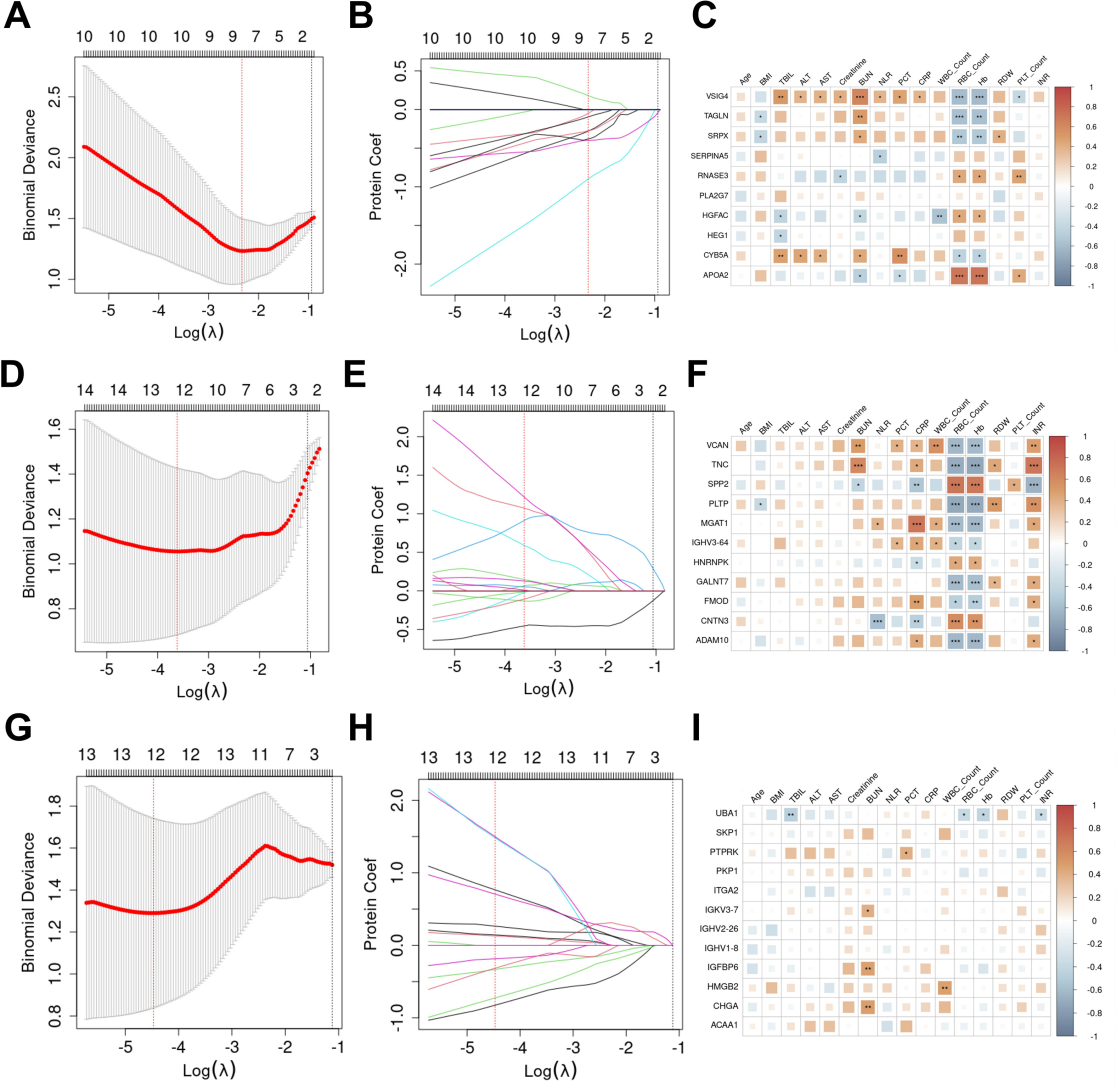
Protein screening. **(A)** Changes in mean squared error (MSE) with Log(λ) during screening of predictive proteins from DEPs between KP Group and Con Group via LASSO regression. The upper horizontal axis was the number of screened proteins. The number of predicted proteins at the minimum mean square error value was the number of proteins screened for subsequent analysis by LASSO regression. **(B)** Changes in regression coefficients with Log(λ) during screening of predictive proteins from KP Group and Con Group via LASSO regression. **(C)** Heatmap of correlations between LASSO-screened proteins (KP group vs. Con group) and clinical variables. Statistical significance was indicated by “*”: "ns" represented not significant (p >0.05), "*" represented significant (0.01< p ≤0.05), "**" represented highly significant (0.001< p ≤0.01), "***" represented extremely significant (p ≤ 0.001). **(D)** Changes in mean squared error (MSE) with Log(λ) during screening of predictive proteins from DEPs between CRKP Group and Con Group via LASSO regression. **(E)** Changes in regression coefficients with Log(λ) during screening of predictive proteins from CRKP Group and Con Group via LASSO regression. **(F)** Heatmap of correlations between LASSO-screened proteins (CRKP group vs. Con group) and clinical variables. **(G)** Changes in mean squared error (MSE) with Log(λ) during screening of predictive proteins from DEPs between CRKP Group and KP Group via LASSO regression. **(H)** Changes in regression coefficients with Log(λ) during screening of predictive proteins from CRKP Group and KP Group via LASSO regression. **(I)** Heatmap of correlations between LASSO-screened key proteins (CRKP group vs. KP group) and clinical variables.

**Figure 3:**
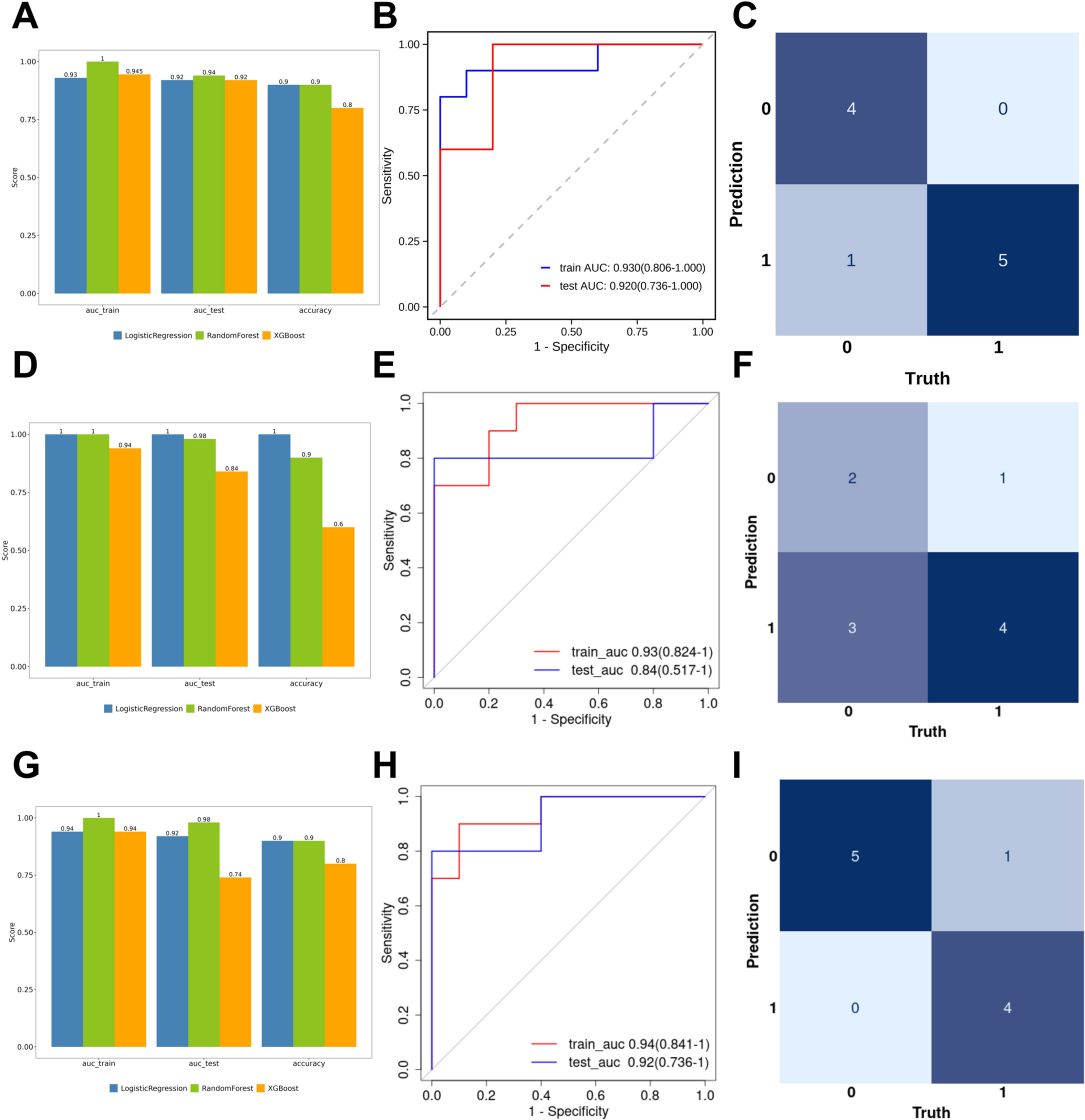
Modeling Results and Evaluation. **(A)** Bar chart of the optimal predictive model performance based on three algorithms for the KP group and the Con group. **(B)** ROC curves of the training set and test set for the KP group and the Con group. The horizontal axis represented 1-specificity, and the vertical axis represented sensitivity. **(C)** Confusion matrix of the prediction results in the test set for the KP group and the Con group. The horizontal axis represented the actual class, and the vertical axis represented the predicted class. **(D)** Bar chart of the optimal predictive model performance based on three algorithms for the CRKP group and the Con group. **(E)** ROC curves of the training set and test set for the CRKP group and the Con group. **(F)** Confusion matrix of the prediction results in the test set for the CRKP group and the Con group. **(G)** Bar chart of the optimal predictive model performance based on three algorithms for the CRKP group and the KP group. **(H)** ROC curves of the training set and test set for the CRKP group and the KP group. **(I)** Confusion matrix of the prediction results in the test set for the CRKP group and the KP group.

To develop diagnostic models for distinguishing CSKP and CRKP infections from controls (Con) and from each other, we employed a consistent machine learning framework across three comparison settings (CSKP vs Con, CRKP vs Con, and CRKP vs CSKP). For each group, 70% of the total samples were allocated to the training set and 30% to the test set. In the training set, Least Absolute Shrinkage and Selection Operator (LASSO) regression with 5-fold cross-validation was applied to identify key proteins from the differentially expressed proteins (DEPs), resulting in 10, 11, and 12 candidate proteins for the three comparisons, respectively. The feature selection process is visually summarized in **Figures 2A, B, D, E, G, H**. Correlation analysis between the selected proteins and clinical parameters revealed VSIG4, VCAN, and UBA1 as the most frequently associated proteins in each group (**Figures 2C, F, I**), showing significant correlations with variables such as RBC count and Hb (P <0.05). Three commonly used algorithms—Logistic Regression, Random Forest, and XGBoost—were then evaluated using 3-fold cross-validation to construct predictive models based on random combinations of the candidate proteins. Models were selected based on the highest test AUC (with a training AUC < 1), with accuracy serving as a tie-breaker. The optimal models were determined as follows: for CSKP vs Con (**Figures 3A–C**; **Supplementary Figures S2A, B**), Logistic Regression with APOA2, HGFAC, and CYB5A (training AUC = 0.930, 95% CI: 0.806–1.000, test AUC = 0.920, 95% CI: 0.736–1.000; accuracy = 90% in both sets; Hosmer–Lemeshow test p-value 0.751 for training set and 0.703 for test set); for CRKP vs Con (**Figures 3D–F**; **Supplementary Figures S2C, D**), XGBoost with CNTN3 and GALNT7 (training AUC = 0.940, 95% CI: 0.819–1.000, test AUC = 0.840, 95% CI: 0.568–1.000; accuracy = 95% and 60%, Hosmer–Lemeshow test p-value 0.421 for training set and 0.092 for test set); and for CRKP vs CSKP (**Figures 3G–I**; **Supplementary Figures S2E, F**), Logistic Regression with IGHV1-8, ITGA2, PKP1, and IGFBP6 (training AUC = 0.940, 95% CI: 0.841–1.000, test AUC = 0.920, 95% CI: 0.736–1.000; accuracy = 90% in both sets, Hosmer–Lemeshow test p-value 0.693 for training set and 0.819 for test set). This protein panel between CRKP and CSKP substantially outperformed individual clinical markers (PCT: 0.640, CRP: 0.560, NLR: 0.520) and the three−marker combination (0.520, 95% CI: 0.070–0.970, **Supplementary Table S3**). To validate the differential expression of modeled proteins, we performed targeted proteomics analysis on an independent validation cohort. All proteins included in the diagnostic model were subjected to PRM measurement. Five of the eight model proteins (CYB5A, APOA2, IGHV1-8, ITGA2, and IGFBP6) were successfully detected and quantified in the validation serum samples (**Supplementary Table S4**). The expression patterns of APOA2 and IGFBP6 were consistent with the discovery cohort and reached statistical significance: compared to the Con group, APOA2 levels were significantly reduced in the CSKP group (p < 0.01; **Supplementary Figure S3A**); IGFBP6 abundance was significantly higher in the CRKP group than in the CSKP group (p < 0.05; **Supplementary Figure S3B**). The AUC value for APOA2 for distinguishing CSKP from the Con group was 0.796 (95% CI: 0.635–0.956, **Supplementary Figure S3C**), while the AUC value for IGFBP6 separating CRKP from CSKP was 0.827 (95% CI: 0.669–0.985, **Supplementary Figure S3D**), demonstrating robust disease discrimination capability for a single protein.

### Severity-associated protein signatures link coagulation, immunity, and metabolic dysfunction in sepsis progression

**Figure 4:**
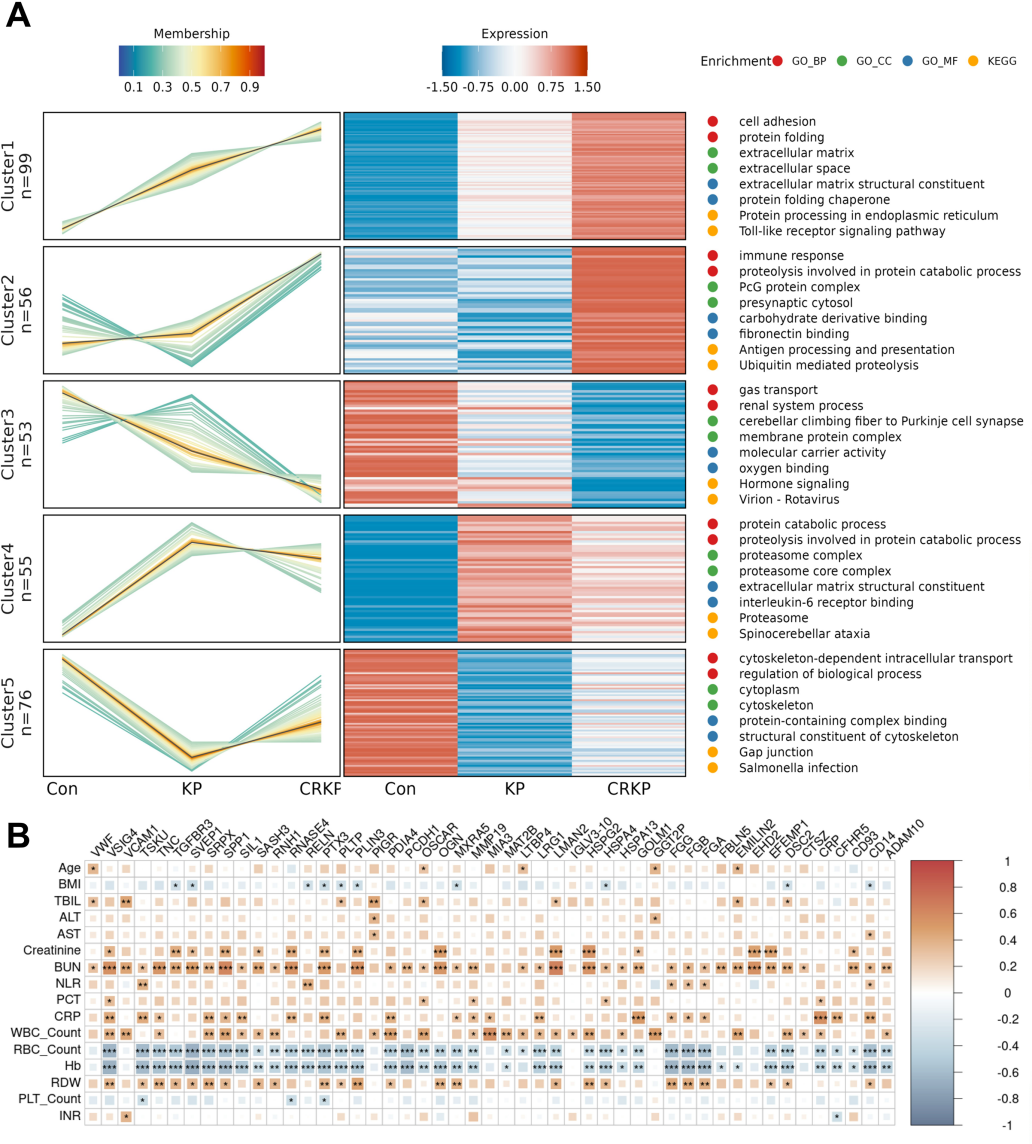
Screening of key proteins by trend clustering analysis. **(A)** Analysis results of Mfuzz clustering. Line graph (left): a protein expression line graph. The horizontal axis represented the sample, the vertical axis depicts the relative protein expression, a line represented a protein, and the color of the line indicated the affiliation intensity in the cluster. Heatmap (middle): the horizontal axis represented the sample, the vertical axis depicted different proteins, and the heatmap color indicated the relative expression of the protein in the sample. The top 2 enrichment analysis entries (right): GO-BP enrichment was red, GO-CC enrichment was green, GO-MF enrichment was blue, and KEGG enrichment was yellow. **(B)** Heatmap of correlations between hub proteins and clinical variables. Only proteins with P-value <0.05 and correlation coefficient >0.3 are shown.

We performed Mfuzz clustering to identify proteins with similar expression patterns across disease groups, revealing five distinct clusters representing different expression profiles (**Figure 4A**). Functional enrichment indicated that these proteins were primarily involved in cell adhesion and protein folding, and were significantly associated with the Toll-like receptor signaling pathway and protein processing in the endoplasmic reticulum. Cluster 1, which initially contained 99 proteins, was refined to 51 high−confidence proteins (unique peptides ≥ 2, membership ≥ 0.4, RSD ≤ 0.4, and p-value <0.05) for further analysis. Correlation heatmap analysis revealed significant associations between Cluster 1 proteins and clinical parameters (**Figure 4B**), with VSIG4, PTX3, and CD14 demonstrating the highest number of significant correlations (p <0.05).

**Figure 5:**
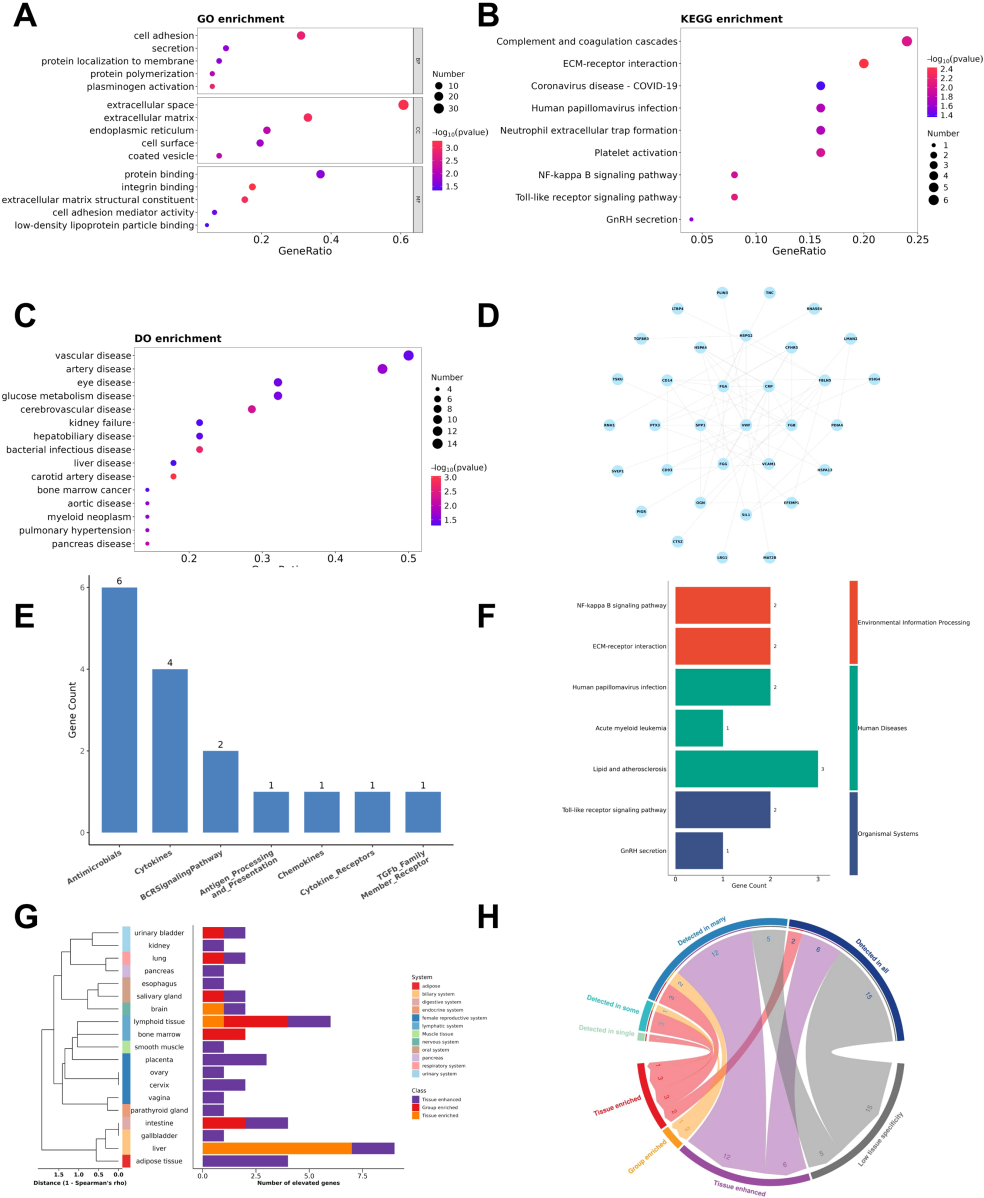
Research on the mechanism of trend proteins. **(A)** GO-based enrichment analysis (two-sided hypergeometric test; P ≤0.05), GO terms were sorted by P-value, and top 5 terms of each category were displayed. **(B)** KEGG-based enrichment analysis (two-sided hypergeometric test; P ≤0.05), KEGG terms were sorted by P-value, and top 15 terms were displayed. **(C)** DO-based enrichment analysis (two-sided hypergeometric test; P ≤0.05), DO terms were sorted by P-value, and top 15 terms were displayed. **(D)** Protein-protein interaction network analysis. Trend proteins were circular. Lines indicated interactions between proteins. **(E)** Bar chart of the immune-related proteins category in trend proteins. **(F)** KEGG pathway enrichment analysis of immune-related proteins. **(G)** (Left) A dendrogram based on the correlation of global expression profiles across all tissues and organs, including blood. (Right) Bar plot displaying the number of hub proteins for each tissue type. **(H)** Chord diagram showing the relationship between the distribution classification and the specificity classification. Each link represented the number of hub proteins in the distribution category and specificity category.

Functional enrichment analysis of Cluster 1 proteins revealed significant involvement in extracellular matrix organization and inflammatory signaling. These proteins were notably enriched in extracellular space and matrix-related cellular components, molecular functions including integrin binding and extracellular matrix structural constituent, and biological processes such as cell adhesion, plasminogen activation, and protein folding (**Figure 5A**). Key KEGG pathways included ECM-receptor interaction, Toll-like receptor signaling and NF-κB signaling, complement and coagulation cascades, and platelet activation (**Figure 5B**). Furthermore, Disease Ontology terms highlighted associations with vascular, hepatobiliary, and inflammatory diseases, as well as bacterial infectious diseases and glucose metabolism disorders (**Figure 5C**), underscoring the cluster’s relevance to systemic host response pathways in sepsis. PPI network analysis of key proteins identified VWF as the hub protein (degree = 11, **Figure 5D**). 16 immune-related proteins, primarily antimicrobials (**Figure 5E**), were enriched in atherosclerosis and ECM-receptor pathways (**Figure 5F**). Tissue specificity analysis revealed the highest protein abundance in liver, with adipose tissue showing maximal tissue-enrichment (**Figures 5G, H**). Drug interaction screening identified five multi-target agents (including ABCIXIMAB and TIROFIBAN) predominantly targeting fibrinogen complexes (**Supplementary Figures S4A, B**). Additional characterization defined transcriptional regulators, kinases, and receptor proteins through specialized databases (**Supplementary Figures S4C–G**).

### Integrated proteomic and metabolomic profiling reveals pathway dysregulation linked to carbapenem resistance

**Figure 6:**
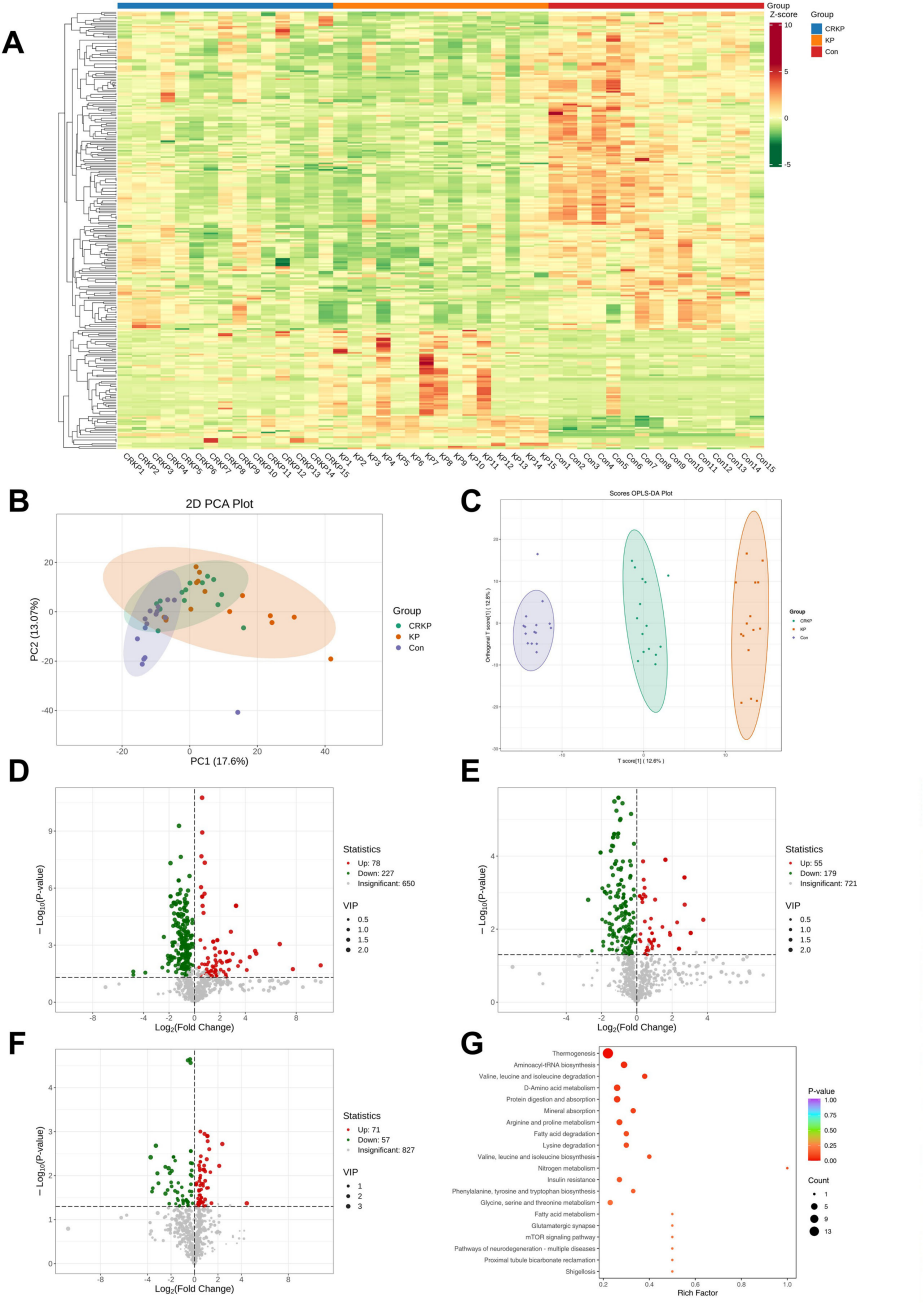
Differential analysis of metabolite abundance levels among KP, CRKP, and Con. **(A)** Cluster Heatmap of DEMs. The horizontal axis represents sample names, and the vertical axis represents differential metabolites. “Group” indicates the sample grouping. Different colors represent the metabolite abundance levels obtained after standardization of different relative contents: red represents high expression, and green represents low expression. **(B)** Principal Component Analysis. PC1 represents the first principal component, and PC2 represents the second principal component. The percentages indicate the explanation rate of each principal component for the dataset. Each dot in the plot represents a sample; samples from the same group are marked with the same color, and "Group" denotes the sample grouping. **(C)** Orthogonal Partial Least Squares-Discriminant Analysis Scores. The horizontal axis represents the predictive principal component, which reflects the differences between groups; the vertical axis represents the orthogonal principal component, which reflects the differences within groups. The percentages indicate the explanation rate of each component for the dataset. Each dot in the plot represents a sample; samples from the same group are marked with the same color, and "Group" denotes the sample grouping. **(D–F)** Volcano plot of DEMs. The horizontal axis represents sample names, and the vertical axis represents differential metabolites. “Group” indicates the sample grouping. Different colors represent the metabolite abundance levels obtained after standardization of different relative contents: red represents high expression, and green represents low expression. **(G)** KEGG enrichment analysis of DEMs between CRKP and CSKP. The horizontal axis represents the Rich Factor corresponding to each pathway, and the vertical axis shows the names of the pathways (sorted by P-value). The color of the dots reflects the magnitude of the P-value: the redder the dot, the more significant the enrichment. The size of the dots represents the number of differential metabolites enriched in the pathway.

Metabolomic profiling revealed distinct patterns among the three groups, with hierarchical clustering showing clear separation patterns in metabolite abundance (**Figure 6A**). PCA demonstrated group segregation (**Figure 6B**), while OPLS-DA models confirmed robust separation between comparison groups (**Figure 6C**). 234 and 307 differentially expressed metabolites (DEMs) were identified when comparing the KP group (CSKP and CRKP) to the Con group, respectively (**Figures 6D, E**). Notably, the CRKP vs. CSKP comparison yielded 128 differential metabolites, with 71 upregulated and 57 downregulated in CRKP (**Figure 6F**). KEGG pathway enrichment analysis demonstrated group-specific metabolic perturbations: KP versus Con DEMs were enriched in mineral absorption, amino acid biosynthesis, and glycerophospholipid metabolism (**Supplementary Figure S5A**); CRKP versus Con DEMs showed alterations in choline metabolism, glycerophospholipid metabolism, and oxidative phosphorylation (**Supplementary Figure S5B**); while CRKP versus CSKP DEMs identified thermogenesis as the most significantly enriched pathway, alongside pronounced alterations in amino acid metabolism (valine, leucine, isoleucine degradation; arginine and proline metabolism) and fatty acid degradation pathways (**Figure 6G**). K-means clustering of all differential metabolites identified across comparison groups revealed six distinct expression patterns (**Supplementary Figure S5C**). Subclass 4, comprising 35 metabolites, exhibited a stepwise increase in abundance across the Con to KP to CRKP groups, suggesting a potential association with disease severity and carbapenem resistance status. Integrated pathway analysis of these stepwise-increased metabolites (Subclass 4) and the severity-associated proteins (Cluster 1) revealed three overlapping KEGG pathways (**Supplementary Figure S8A**). Among these, cysteine and methionine metabolism (**Supplementary Figure S6**) and one carbon pool by folate (**Supplementary Figure S7**) were significantly enriched (p < 0.05) (**Supplementary Figure S8B**). Notably, MAT2B emerged as the shared protein linking both pathways and exhibited significantly altered abundance between the Con and CRKP groups (**Supplementary Figure 8C**).

## Discussion

This study, for the first time, from a host systems biology perspective, comprehensively revealed the serum proteomic and metabolomic characteristics of sepsis patients with CRKP infection. Our findings indicated that host molecular features related to coagulation, immune, and metabolic disorders are markedly elevated in CRKP infections. We identified multiple host-derived protein biomarker panels with high diagnostic performance, enabling effective discrimination of CRKP infection from CSKP infection and culture-negative controls. These results offer a preliminary foundation for developing early, minimally invasive, and precise diagnostic tools for CRKP infection, with the potential to support timely targeted clinical interventions.

85 DEPs differentiating CRKP from CSKP were enriched in pathways related to viral infection, antigen processing and presentation, and phagosome function. It is important to note that the enrichment of ‘viral infection’ pathways (e.g., HIV-1 infection, Cytomegalovirus infection) does not suggest viral co-pathogenesis. Instead, these KEGG pathway maps represent fundamental immune signaling cascades—including type I interferon responses, endosomal TLR signaling, and MHC class I antigen presentation—that are also potently activated during severe bacterial sepsis. The enrichment of these pathways in CRKP relative to CSKP suggests a more pronounced or dysregulated activation of these innate immune modules in response to the drug-resistant pathogen. This pattern suggests that CRKP infection may elicit a host immune response bearing hallmarks typically observed in viral infections, potentially involving aberrant interferon signaling or altered antigen presentation dynamics. Furthermore, the enrichment of phosphatidylinositol and calcium signaling pathways underscores a profound rewiring of intracellular signaling networks in response to drug-resistant infection. PPI network analysis consolidated these findings, revealing a highly interconnected module with CD44 and B2M emerging as top hub proteins. CD44, a multifunctional cell-surface receptor (39), is critically involved in immune cell activation, trafficking, and inflammatory responses to bacterial challenge (40). Its central position suggests a role in modulating leukocyte recruitment and intercellular communication within the dysregulated tissue microenvironment during CRKP sepsis (41). Concurrently, B2M, an essential component of the major histocompatibility complex (MHC) class I, is indispensable for peptide antigen presentation to T lymphocytes (42). Its hub status indicates that alterations in MHC-I-mediated antigen presentation may be a pivotal mechanism underpinning the immune response to CRKP, possibly mirroring immune evasion strategies documented in certain viral infections and mycobacterial pathogens. Thus, the coordinated prominence of CD44 and B2M highlights a concerted dysregulation of both innate sensing and adaptive immune recognition in CRKP sepsis, potentially contributing to the inadequate pathogen clearance and immune dysfunction that characterize these infections.

The Mfuzz trend analysis delineated a cluster of 51 proteins (Cluster 1) exhibiting a stepwise increase in abundance from control sepsis to CSKP and further to CRKP infection. Functional annotation revealed that these proteins were significantly enriched in biological processes and pathways central to the host systemic immune response, including cell adhesion, Toll-like receptor signaling, and protein processing in the endoplasmic reticulum. This expression gradient observed across the groups suggests a stepwise escalation of endothelial activation, innate immune signaling, and ER stress correlating with both the infection presence and, more profoundly, with carbapenem resistance. The PPI network consolidated these findings, pinpointing VWF as the predominant hub node. VWF, a critical mediator of hemostasis, was integral to multiple KEGG pathways enriched in our dataset, such as PI3K-Akt signaling pathway, ECM-receptor interaction, complement and coagulation cascades, and platelet activation. Its central role underscored a pathophysiological axis where dysregulated coagulation, inflammation, and endothelial dysfunction are synergistically amplified in CRKP sepsis. Emerging evidence has implicated VWF in exacerbating endothelial hyperpermeability and contributing to microthrombus formation in vital organs during sepsis (43). The marked upregulation of VWF across our patient cohorts, culminating in CRKP infection, aligned with clinical observations linking high VWF levels and an imbalance with its cleaving protease ADAMTS13 to disease severity and disseminated intravascular coagulation (44). Thus, VWF occupies a central position within this severity associated protein module, highlighting its pivotal role in the immunothrombotic cascade. This finding also suggests that the VWF pathway and its regulatory mechanisms represent potential therapeutic targets—inhibition of this pathway may mitigate organ injury and improve outcomes in CRKP sepsis.

There remains a clinical need for the rapid discrimination between CRKP and CSKP infections, as early and accurate antibiotic selection is critical for improving patient outcomes. The current gold standard, relying on blood culture and antimicrobial susceptibility testing, typically entails a delay of 48–72 hours, potentially creating a window period for empirical treatment (45). Prediction models based on clinical features or pathogen genotypes are often limited by suboptimal specificity. In this context, we developed a four-protein host-derived biomarker panel (IGHV1−8, ITGA2, PKP1, IGFBP6) that demonstrated high diagnostic performance in both training and test sets (AUC = 0.940 and 0.920, respectively), substantially outperforming conventional single biomarkers. Furthermore, using PRM, a targeted mass spectrometry method, we independently validated key proteins in a separate cohort, confirming the significant upregulation of IGFBP6 in CRKP compared with CSKP patients, as well as the differential expression of APOA2 in CSKP versus culture−negative controls. Previous studies have revealed that IGFBP6 binds to PHB2 on epithelial cells in an IGF−independent manner during sepsis, inhibiting the STAT1−CCL2 axis and thereby impairing macrophage chemotaxis and bactericidal function, suggesting its potential role as a key regulator in sepsis progression (46). Similarly, APOA2 has been identified through multi−omics and genetic analyses as a protective biomarker for sepsis-related lung injury, with its expression level showing a negative relationship with sepsis risk (47). These findings not only support the biological plausibility of the alterations in IGFBP6 and APOA2 observed in our cohort but also indicate that they may participate in distinct host immune−metabolic dysregulation processes in the context of CRKP versus CSKP infections. Our subsequent research will focus on large-scale prospective validation, translating findings into high-throughput clinical testing protocols, and investigating the functional roles of IGFBP6 and APOA2 in the pathophysiology of drug-resistant sepsis through animal or cellular models.

The findings of this study hold potential relevance for emergency and critical care practice. Early differentiation of CRKP from CSKP infection remains challenging at the bedside, and current microbiological diagnostics impose a critical delay that often necessitates broad-spectrum empirical therapy. A host-based proteomic signature, if further validated in larger prospective studies, could be developed into a rapid test to assist ED and ICU clinicians in early risk stratification and more targeted antimicrobial selection (18, 48). Such an approach would complement existing clinical judgment and biomarkers, with the goal of narrowing the window of inappropriate therapy. In addition to the ED setting, this host−based multi−omics diagnostic strategy also has potential applicability in other high−risk clinical scenarios, including intensive care units (ICUs), hematology departments, and solid organ transplant wards, where CRKP infection is highly prevalent and associated with poor outcomes (49, 50). The core molecular pathways identified in this study, such as cysteine/methionine metabolism and folate−mediated one−carbon pool metabolism, as well as key proteins including MAT2B, VWF and IGFBP6, may also serve as potential targets for adjuvant therapy in acute infection, providing new ideas for immunometabolic regulation and precision intervention beyond antimicrobial treatment. With further large−scale prospective validation and the development of rapid detection kits, this biomarker panel is expected to be integrated into routine ED testing processes, forming a complementary system with traditional microbiological methods to improve the early diagnosis and acute management level of drug−resistant bacterial sepsis.Integrated pathway analysis revealed two KEGG pathways with significant overlap between progressively upregulated metabolites and proteins: cysteine and methionine metabolism, and one-carbon pool by folate. Dysregulation of the folate-driven one-carbon pool can lead to global DNA hypomethylation, a state associated with genomic instability and DNA strand breaks, potentially contributing to the widespread cellular dysfunction and organ damage observed in severe sepsis (51). Furthermore, previous studies have suggested that methionine supplementation may suppress the transcription and translation of resistance genes in pathogenic bacteria, thereby enhancing antibiotic efficacy, further highlighting the central role of methionine metabolism in research on drug-resistant pathogens (52). Among these pathways, MAT2B emerged as a key protein, showing a significantly elevated abundance in CRKP patients. MAT2B is the regulatory subunit of methionine adenosyltransferase (MAT), which forms a complex with the catalytic subunit MAT2A to modulate S−adenosylmethionine (SAM) synthesis, rather than directly catalysing it (53). MAT2B has been implicated in various cancers (54, 55), including non-small cell lung cancer (56), and its elevated expression has been associated with chemosensitivity to multiple drugs (57). In our study, MAT2B was significantly upregulated in CRKP patients compared to controls, suggesting a potential link between one−carbon metabolism and the host response to carbapenem−resistant infection. However, we caution that these findings are purely observational and do not establish a causal role for MAT2B in disease pathogenesis or drug resistance. The regulatory function of MAT2B within the MAT complex implies that its effects are likely mediated through modulation of MAT2A activity and subsequent SAM production, but this hypothesis requires direct experimental testing. Future studies using cellular or animal models with targeted manipulation of MAT2B expression, combined with functional assays of one−carbon metabolism and antimicrobial susceptibility, are needed before any translational implications can be considered.

This study has several limitations that should be considered. First, the cohort was recruited from a limited number of hospitals in China with a relatively small sample size, which may restrict the statistical power and generalizability of the findings. No formal sample size calculation was performed for the multi-omics or machine learning analyses, as this was an exploratory discovery study without pre-defined effect sizes. Consequently, the statistical power may be limited, and the reported diagnostic performance metrics require independent validation in larger cohorts. Potential selection bias cannot be excluded, and further prospective, multi-center studies involving diverse ethnic populations are needed to validate the diagnostic model and biomarker panels. Second, given the limited sample size and exploratory nature of the study, we employed a discovery threshold of p<0.05 without multiple comparison correction (e.g., false discovery rate) to maximize sensitivity in identifying candidate markers. This decision aimed to avoid overlooking potentially relevant biological signals, though we acknowledge this approach increases the risk of false positives. Third, DEPs were selected from the full discovery dataset before splitting, which may introduce information leakage. However, given the small sample size (n = 15 per group) in this exploratory study, performing DEP identification only on the training subset would drastically reduce statistical power, leading to unstable and irreproducible feature selection. Fourth, the use of the test set for model selection in our machine learning analysis may have resulted in overoptimistic performance estimates. Given the small sample size, nested cross−validation was not feasible. Consequently, the candidate protein panel should be considered exploratory, and its reported AUC values require independent validation in larger cohorts. Fifth, we did not record SOFA scores, vasopressor use, lactate levels, or other detailed illness severity variables. Therefore, we cannot fully disentangle the effects of carbapenem resistance from disease severity. Future prospective studies should systematically collect these variables for more rigorous confounding control. Sixth, while our multi-omics approach provides a comprehensive host molecular landscape, the identified differential proteins and metabolites may not be specific to CRKP infection but could reflect broader sepsis-related host responses or organ dysfunction. More targeted experimental validation is required to confirm their specific roles in resistance mechanisms. Seventh, no joint analysis of MAT2A and MAT2B was performed. However, MAT2A could not be quantified due to excessive missing values (>70%) and was therefore excluded from analysis. This reflects the challenge of detecting low−abundance intracellular proteins in serum. Additionally, Haemolysis indices were not systematically assessed. Although visibly haemolysed samples were excluded by visual inspection, mild sub−haemolytic states may still affect quantification of certain analytes. Future studies should routinely measure haemolysis markers. Furthermore, the specific quantification of immunoglobulin variable region proteins (e.g., IGHV1-8) in serum is complicated by sequence homology; results for IGHV1–8 should be interpreted with caution, their reliability as biomarkers should be further validated by immuno-based methods. Finally, while we highlighted key pathways and molecules such as MAT2B, VWF, and IGFBP6, further functional studies are necessary to elucidate their precise mechanisms in CRKP pathogenesis and to explore their potential as therapeutic targets. Future research should also integrate longitudinal sampling to track dynamic host responses and assess the prognostic value of the identified signatures.

## Conclusion

In summary, this study combines proteomic and metabolomic profiling to delineate the host serum molecular landscape of sepsis caused by CRKP. Our work suggests distinct molecular signatures associated with carbapenem resistance, including a severity-associated protein cluster linked to coagulation, immune signaling, and metabolic dysfunction. The identified four−protein biomarker panel (IGHV1−8, ITGA2, PKP1, IGFBP6) effectively discriminates CRKP from CSKP infection. Integrated pathway analysis points to potential dysregulation in cysteine and methionine metabolism, and one carbon pool by folate, with MAT2B emerging as a key nodal protein. Although limited by sample size and single−center design, these findings provide a host−centered molecular foundation for early, non−invasive diagnosis and precision intervention strategies in CRKP sepsis. Further validation in larger cohorts and functional studies on highlighted proteins such as MAT2B, VWF, and IGFBP6 will be essential to translate these insights into clinical practice.

## Data Availability

The mass spectrometry proteomics data have been deposited to the ProteomeXchange Consortium via the iProx partner repository with the dataset identifier PXD075261. The metabolomics data have been deposited to the MetaboLights database with the identifier MTBLS14016.

## References

[1] Singer M Deutschman CS Seymour CW Shankar-Hari M Annane D Bauer M . The third international consensus definitions for sepsis and septic shock (Sepsis-3). Jama. (2016) 315:801–10. doi:10.1001/jama.2016.0287. PMID: 26903338 PMC4968574

[2] Rudd KE Johnson SC Agesa KM Shackelford KA Tsoi D Kievlan DR . Global, regional, and national sepsis incidence and mortality, 1990–2017: analysis for the Global Burden of Disease Study. Lancet. (2020) 395:200–11. doi:10.1016/s0140-6736(19)32989-7. PMID: 31954465 PMC6970225

[3] Angus DC van der Poll T . Severe sepsis and septic shock. N Engl J Med. (2013) 369:840–51. doi:10.1056/nejmra1208623. PMID: 23984731

[4] Chen J Ma H Huang X Cui Y Peng W Zhu F . Risk factors and mortality of carbapenem-resistant Klebsiella pneumoniae bloodstream infection in a tertiary-care hospital in China: an eight-year retrospective study. Antimicrobial Resistance Infection Control. (2022) 11(1):161. doi:10.21203/rs.3.rs-134806/v1. PMID: 36536423 PMC9761986

[5] Cheng Y Cheng Q Zhang R Gao J Li W Wang F . Retrospective analysis of molecular characteristics, risk factors, and outcomes in carbapenem-resistant Klebsiella pneumoniae bloodstream infections. BMC Microbiol. (2024) 24(1):309. doi:10.1186/s12866-024-03465-4. PMID: 39174950 PMC11340057

[6] Chen Y Huang HB Peng JM Weng L Du B . Efficacy and safety of ceftazidime-avibactam for the treatment of carbapenem-resistant Enterobacterales bloodstream infection: a systematic review and meta-analysis. Microbiol Spectr. (2022) 10:e0260321. doi:10.1128/spectrum.02603-21. PMID: 35377233 PMC9045088

[7] Wang G Cao L Lian L Wang Y Lian J Liu Z . Machine learning and DIA proteomics reveal new insights into carbapenem resistance mechanisms in Klebsiella pneumoniae. J Proteome Res. (2025) 24:4002–14. doi:10.1021/acs.jproteome.5c00142. PMID: 40622342

[8] Meekes LM Heikema AP Tompa M Astorga Alsina AL Hiltemann SD Stubbs AP . Proteogenomic analysis demonstrates increased bla(OXA-48) copy numbers and OmpK36 loss as contributors to carbapenem resistance in Klebsiella pneumoniae. Antimicrob Agents Chemother. (2025) 69:e0010725. doi:10.1128/aac.00107-25. PMID: 40512052 PMC12217476

[9] Cox PB Teo J-M Fouts DE Clarke TH Ruffin F Fowler VG . Molecular epidemiology and clinical characteristics of carbapenem-resistant Klebsiella pneumoniae bloodstream and pneumonia isolates. Microbiol Spectr. (2025) 13:e0063125. doi:10.1128/spectrum.00631-25. PMID: 40631745 PMC12323367

[10] Queiroz PA Meneguello JE Silva BR Caleffi-Ferracioli KR Scodro RB Cardoso RF . Proteomic profiling of Klebsiella pneumoniae carbapenemase (KPC)-producer Klebsiella pneumoniae after induced polymyxin resistance. Future Microbiol. (2021) 16:1195–207. doi:10.2217/fmb-2021-0005. PMID: 34590903

[11] Hussein M Jasim R Gocol H Baker M Thombare VJ Ziogas J . Comparative proteomics of outer membrane vesicles from polymyxin-susceptible and extremely drug-resistant Klebsiella pneumoniae. mSphere. (2023) 8:e0053722. doi:10.1128/msphere.00537-22. PMID: 36622250 PMC9942579

[12] Butler JM Peters-Sengers H Reijnders TDY van Engelen TSR Uhel F van Vught LA . Pathogen-specific host response in critically ill patients with blood stream infections: a nested case–control study. EBioMedicine. (2025) 117:105799. doi:10.1016/j.ebiom.2025.105799. PMID: 40505417 PMC12182767

[13] Wu T Xu F Su C Li H Lv N Liu Y . Alterations in the gut microbiome and cecal metabolome during Klebsiella pneumoniae-induced pneumosepsis. Front Immunol. (2020) 11:1331. doi:10.3389/fimmu.2020.01331. PMID: 32849494 PMC7411141

[14] Aye SM Galani I Han M-L Karaiskos I Creek DJ Zhu Y . Lipid A profiling and metabolomics analysis of paired polymyxin-susceptible and -resistant MDR Klebsiella pneumoniae clinical isolates from the same patients before and after colistin treatment. J Antimicrob Chemother. (2020) 75:2852–63. doi:10.1093/jac/dkaa245. PMID: 32696049 PMC8453395

[15] Dixon B Ahmed WM Fowler SJ Felton T Trivedi DK . LC-MS/MS metabolomics unravels the resistant phenotype of carbapenemase-producing Enterobacterales. Metabolomics. (2025) 21(5):115. doi:10.1007/s11306-025-02300-9. PMID: 40794220 PMC12343662

[16] van der Poll T Shankar-Hari M Wiersinga WJ . The immunology of sepsis. Immunity. (2021) 54:2450–64. doi:10.1016/j.immuni.2021.10.012. PMID: 34758337

[17] Joosten SCM Wiersinga WJ Poll T . Dysregulation of host–pathogen interactions in sepsis: host-related factors. Semin Respir Crit Care Med. (2024) 45:469–78. doi:10.1055/s-0044-1787554. PMID: 38950605 PMC11663080

[18] Turgman O Schinkel M Wiersinga WJ . Host response biomarkers for sepsis in the emergency room. Crit Care (London England). (2023) 27:97. doi:10.1007/978-3-031-23005-9_6. PMID: 36941681 PMC10027585

[19] Batth TS Francavilla C Olsen JV . Off-line high-pH reversed-phase fractionation for in-depth phosphoproteomics. J Proteome Res. (2014) 13:6176–86. doi:10.1021/pr500893m. PMID: 25338131

[20] Meier F Brunner AD Koch S Koch H Lubeck M Krause M . Online parallel accumulation-serial fragmentation (PASEF) with a novel trapped ion mobility mass spectrometer. Mol Cell Proteomics: MCP. (2018) 17:2534–45. doi:10.1074/mcp.tir118.000900. PMID: 30385480 PMC6283298

[21] Meier F Brunner AD Frank M Ha A Bludau I Voytik E . diaPASEF: parallel accumulation-serial fragmentation combined with data-independent acquisition. Nat Methods. (2020) 17:1229–36. doi:10.1038/s41592-020-00998-0. PMID: 33257825

[22] Ahmad S Jose da Costa Gonzales L Bowler-Barnett EH Rice DL Kim M Wijerathne S . The UniProt website API: facilitating programmatic access to protein knowledge. Nucleic Acids Res. (2025) 53:W547–53. doi:10.1093/nar/gkaf394. PMID: 40331428 PMC12230682

[23] Cox J Hein MY Luber CA Paron I Nagaraj N Mann M . Accurate proteome-wide label-free quantification by delayed normalization and maximal peptide ratio extraction, termed MaxLFQ. Mol Cell Proteomics: MCP. (2014) 13:2513–26. doi:10.1074/mcp.m113.031591. PMID: 24942700 PMC4159666

[24] Saleh S Staes A Deborggraeve S Gevaert K . Targeted proteomics for studying pathogenic bacteria. Proteomics. (2019) 19:e1800435. doi:10.1002/pmic.201800435. PMID: 31241236

[25] Chen W Gong L Guo Z Wang W Zhang H Liu X . A novel integrated method for large-scale detection, identification, and quantification of widely targeted metabolites: application in the study of rice metabolomics. Mol Plant. (2013) 6:1769–80. doi:10.1093/mp/sst080. PMID: 23702596

[26] Eriksson L Kettanehwold N Trygg J Wikström C Wold SJUI . Multi- and megavariate data analysis: part I: basic principles and applications. (2006). Eriksson L, Kettanehwold N, Trygg J, Wikström C, Wold SJUI. Multi- and Megavariate Data Analysis: Part I: Basic Principles and Applications. 2006.

[27] Thévenot EA Roux A Xu Y Ezan E Junot C . Analysis of the human adult urinary metabolome variations with age, body mass index, and gender by implementing a comprehensive workflow for univariate and OPLS statistical analyses. J Proteome Res. (2015) 14:3322–35. doi:10.1007/978-3-319-53444-2_8. PMID: 26088811

[28] Aleksander SA Balhoff J Carbon S Cherry JM Drabkin HJ Ebert D . The gene ontology knowledgebase in 2023. Genetics. (2023) 224(1):iyad031. doi:10.1093/genetics/iyad031. PMID: 36866529 PMC10158837

[29] Kanehisa M Goto S . KEGG: kyoto encyclopedia of genes and genomes. Nucleic Acids Res. (2000) 28:27–30. doi:10.1093/nar/28.1.27. PMID: 10592173 PMC102409

[30] Schriml LM Arze C Nadendla S Chang YW Mazaitis M Felix V . Disease Ontology: a backbone for disease semantic integration. Nucleic Acids Res. (2012) 40:D940–6. doi:10.1093/nar/gkr972. PMID: 22080554 PMC3245088

[31] Szklarczyk D Kirsch R Koutrouli M Nastou K Mehryary F Hachilif R . The STRING database in 2023: protein-protein association networks and functional enrichment analyses for any sequenced genome of interest. Nucleic Acids Res. (2023) 51:D638–46. doi:10.1093/nar/gkac1000. PMID: 36370105 PMC9825434

[32] Pang Z Lu Y Zhou G Hui F Xu L Viau C . MetaboAnalyst 6.0: towards a unified platform for metabolomics data processing, analysis and interpretation. Nucleic Acids Res. (2024) 52:W398–406. doi:10.1093/nar/gkae253. PMID: 38587201 PMC11223798

[33] Bhattacharya S Dunn P Thomas CG Smith B Schaefer H Chen J . ImmPort, toward repurposing of open access immunological assay data for translational and clinical research. Sci Data. (2018) 5:180015. doi:10.1038/sdata.2018.15. PMID: 29485622 PMC5827693

[34] Uhlen M Oksvold P Fagerberg L Lundberg E Jonasson K Forsberg M . Towards a knowledge-based human protein atlas. Nat Biotechnol. (2010) 28:1248–54. doi:10.1038/nbt1210-1248. PMID: 21139605

[35] Cannon M Stevenson J Stahl K Basu R Coffman A Kiwala S . DGIdb 5.0: rebuilding the drug-gene interaction database for precision medicine and drug discovery platforms. Nucleic Acids Res. (2024) 52:D1227–35. doi:10.1093/nar/gkad1040. PMID: 37953380 PMC10767982

[36] Chen T Guestrin C . (2016). “ XGBoost: a scalable tree boosting system”, in: Proceedings of the 22nd ACM SIGKDD International Conference on Knowledge Discovery and Data Mining (San Francisco, California, USA: Association for Computing Machinery), 785–94.

[37] Alba AC Agoritsas T Walsh M Hanna S Iorio A Devereaux PJ . Discrimination and calibration of clinical prediction models: users’ guides to the medical literature. Jama. (2017) 318:1377–84. doi:10.1001/jama.2017.12126. PMID: 29049590

[38] Chong J Xia J . MetaboAnalystR: an R package for flexible and reproducible analysis of metabolomics data. Bioinf (Oxford England). (2018) 34:4313–4. doi:10.1093/bioinformatics/bty528. PMID: 29955821 PMC6289126

[39] Crosby HA Lalor PF Ross E Newsome PN Adams DH . Adhesion of human haematopoietic (CD34+) stem cells to human liver compartments is integrin and CD44 dependent and modulated by CXCR3 and CXCR4. J Hepatol. (2009) 51:734–9. doi:10.1016/j.jhep.2009.06.021. PMID: 19703720

[40] Funaro A Spagnoli GC Momo M Knapp W Malavasi F . Stimulation of T cells via CD44 requires leukocyte-function-associated antigen interactions and interleukin-2 production. Hum Immunol. (1994) 40:267–78. doi:10.1016/0198-8859(94)90026-4. PMID: 7528188

[41] Casalino-Matsuda SM Monzon ME Day AJ Forteza RM . Hyaluronan fragments/CD44 mediate oxidative stress-induced MUC5B up-regulation in airway epithelium. Am J Respir Cell Mol Biol. (2009) 40:277–85. doi:10.1165/rcmb.2008-0073oc. PMID: 18757307 PMC2645525

[42] Sreejit G Ahmed A Parveen N Jha V Valluri VL Ghosh S . The ESAT-6 protein of Mycobacterium tuberculosis interacts with beta-2-microglobulin (β2M) affecting antigen presentation function of macrophage. PloS Pathog. (2014) 10:e1004446. doi:10.1371/journal.ppat.1004446. PMID: 25356553 PMC4214792

[43] Liu YS Chen WL Zeng YW Li ZH Zheng HL Pan N . Isaridin E protects against sepsis by inhibiting Von Willebrand factor-induced endothelial hyperpermeability and platelet-endothelium interaction. Mar Drugs. (2024) 22(6):283. doi:10.3390/md22060283. PMID: 38921594 PMC11204489

[44] Peetermans M Meyers S Liesenborghs L Vanhoorelbeke K De Meyer SF Vandenbriele C . Von Willebrand factor and ADAMTS13 impact on the outcome of Staphylococcus aureus sepsis. J Thromb Haemostasis: JTH. (2020) 18:722–31. doi:10.1111/jth.14686. PMID: 31758651

[45] Seymour CW Liu VX Iwashyna TJ Brunkhorst FM Rea TD Scherag A . Assessment of clinical criteria for sepsis: for the third international consensus definitions for sepsis and septic shock (Sepsis-3). Jama. (2016) 315:762–74. doi:10.1001/jama.2016.0287. PMID: 26903335 PMC5433435

[46] Chen K Hu Y Yu X Tang H Ruan Y Li Y . IGFBP6 orchestrates antiinfective immune collapse in murine sepsis via prohibitin-2-mediated immunosuppression. J Clin Invest. (2025) 135(21):e184721. doi:10.1172/jci184721. PMID: 40892465 PMC12578393

[47] Wang Y Fan Y Jiang Y Wang E Song Y Chen H . APOA2: new target for molecular hydrogen therapy in sepsis-related lung injury based on proteomic and genomic analysis. Int J Mol Sci. (2023) 24(14):11325. doi:10.3390/ijms241411325. PMID: 37511084 PMC10379236

[48] DuChateau B Murphy S Tarr C Gottlieb T Spies S . B-047 A rapid host-response test supports antimicrobial stewardship at a micro-hospital emergency department. Clin Chem. (2024) 70. doi:10.1093/clinchem/hvae106.409

[49] Girmenia C Viscoli C Piciocchi A Cudillo L Botti S Errico A . Management of carbapenem resistant Klebsiella pneumoniae infections in stem cell transplant recipients: an Italian multidisciplinary consensus statement. Haematologica. (2015) 100:e373–6. doi:10.3324/haematol.2015.125484. PMID: 25862702 PMC4800687

[50] Giannella M Bartoletti M Conti M Righi E . Carbapenemase-producing Enterobacteriaceae in transplant patients. J Antimicrob Chemother. (2021) 76:i27–39. doi:10.1093/jac/dkaa495. PMID: 33534881

[51] Xu X Chen J . One-carbon metabolism and breast cancer: an epidemiological perspective. J Genet Genomics = Yi Chuan Xue Bao. (2009) 36:203–14. doi:10.1016/s1673-8527(08)60108-3. PMID: 19376481 PMC2694962

[52] Fang D Xu T Li F Sun Y Sun J Yin Y . Methionine-driven methylation modification overcomes plasmid-mediated high-level tigecycline resistance. Nat Commun. (2025) 16:417. doi:10.1038/s41467-024-55791-w. PMID: 39762254 PMC11704046

[53] Murray B Antonyuk SV Marina A Van Liempd SM Lu SC Mato JM . Structure and function study of the complex that synthesizes S-adenosylmethionine. IUCrJ. (2014) 1:240–9. doi:10.1007/978-3-030-28151-9_15. PMID: 25075345 PMC4107924

[54] Xu J Wu D Wang S Wang Z . MAT2B expression correlates with poor prognosis in triple-negative breast cancer. Cancer Manage Res. (2019) 11:5501–11. doi:10.2147/cmar.s200716. PMID: 31354356 PMC6585407

[55] Zhang C Lu X Ni T Wang Q Gao X Sun X . Developing patient-derived organoids to demonstrate JX24120 inhibits SAMe synthesis in endometrial cancer by targeting MAT2B. Pharmacol Res. (2024) 209:107420. doi:10.1016/j.phrs.2024.107420. PMID: 39293586

[56] Wang H Feng L Cheng D Zheng Y Xie Y Fu B . Circular RNA MAT2B promotes migration, invasion and epithelial-mesenchymal transition of non-small cell lung cancer cells by sponging miR-431. Cell Cycle (Georgetown Tex). (2021) 20:1617–27. doi:10.1080/15384101.2021.1956106. PMID: 34288814 PMC8409757

[57] Min D-J Vural S Krushkal J . Association of transcriptional levels of folate-mediated one-carbon metabolism-related genes in cancer cell lines with drug treatment response. Cancer Genet. (2019) 237:19–38. doi:10.1016/j.cancergen.2019.05.005. PMID: 31447063

