# Supplementary material for: Serum proteomic profiling of sepsis patients reveals a protein-based diagnostic model, with metabolomic insights into carbapenem-resistant Klebsiella pneumoniae infection
Source: Front Immunol. 2026 May 20;17:1818068. doi: 10.3389/fimmu.2026.1818068 (PMC13230049; doi:10.3389/fimmu.2026.1818068)

**Supplementary materials**

**Serum Proteomic Profiling of Sepsis Patients Reveals a Protein-Based Diagnostic Model, with Metabolomic Insights into Carbapenem-Resistant Klebsiella pneumoniae Infection**

Juan He ^1,7^, Siqi Luo ^2,7^, Wenyun Xu ^1,7^, Yuanzhuo Chen ^3^, Guorong Liu ^4^, Jianguo Tang ^5^, Yong Yang ^2^, Bing Zhao ^6, *^, Li Ma ^6, *^, Huiqiu Sheng ^6, *^, Enqiang Mao ^6, *^

^1^ Department of Pharmacy, Ruijin Hospital, Shanghai Jiao Tong University School of Medicine, Shanghai, 200025, China.

^2^ SpecAlly Life Technology Co., Ltd., Wuhan, China.

^3^ Department of Emergency, the Tenth People’s Hospital, Tongji University, Shanghai 200072, China.

^4^ Department of Emergency, Gongli Hospital of Shanghai Pudong New Area, Shanghai 200135, China.

^5^ Department of Emergency, the Fifth People's Hospital of Shanghai, Fudan University, Shanghai 201100, China.

^6^ Department of Emergency, Ruijin Hospital, Shanghai Jiao Tong University School of Medicine, Shanghai, 200025, China.

^7^ Juan He, Siqi Luo and Wenyun Xu are co-first authors who have contributed equally to this work.

- Corresponding authors:

Dr. Enqiang Mao, Department of Emergency, Ruijin Hospital, Shanghai Jiao Tong University School of Medicine, Shanghai, 200025, China. Email: [maoeq@yeah.net](mailto:maoeq@yeah.net).

Dr. Huiqiu Sheng, Department of Emergency, Ruijin Hospital, Shanghai Jiao Tong University School of Medicine, Shanghai, 200025, China. Email: shenghq803@163.com.

Dr. Li Ma, Department of Emergency, Ruijin Hospital, Shanghai Jiao Tong University School of Medicine, Shanghai, 200025, China. Email: [malipostgraduate2@163.com](mailto:malipostgraduate2@163.com).

Dr. Bing Zhao, Department of Emergency, Ruijin Hospital, Shanghai Jiao Tong University School of Medicine, Shanghai, 200025, China. Email: zhaobing124@163.com.

**Table of contents**

**Supplementary Methods:** Proteomic Data Quality Control

**Table S1** Isolation list for PRM standard mode.

**Table S2** Descriptive characteristics of validation cohort.

**Table S3** Performance of the candidate protein panel and clinical markers in distinguishing sepsis groups.

**Table S4** Targeted PRM quantification and differential abundance analysis of model-derived proteins in the independent validation cohort.

**Figure S1** Population Characteristics and Data Overview.

**Figure S2** Calibration curves and decision curves for the final models in the test set.

**Figure S3** Validation of APOA2 and IGFBP6 expression patterns and diagnostic performance in the validation cohort.

**Figure S4** Drug-protein interaction network of trend proteins.

**Figure S5** Enrichment analysis and cluster analysis of DEMs.

**Figure S6** Schematic diagram of the One carbon pool by folate pathway**.**

**Figure S7** Schematic diagram of the Cysteine and methionine metabolism pathway.

**Figure S8** KEGG pathway integrated analysis of proteomics and metabolomics.

****Supplementary Methods:** Proteomic Data Quality Control**

A total of 1434 proteins and 11802 peptides were quantified across all samples (Figures S1A and 1B). The distribution of peptide lengths showed a predominant range of 7–20 amino acids, consistent with effective tryptic digestion (Figure S1C). The majority of proteins were identified by multiple peptides, with over 81% of the proteins quantified by at least two unique peptides, supporting robust protein identification reliability (Figure S1D). Analysis of missed cleavage sites revealed that more than 76% of peptides contained no missed cleavages (Figure S1E). The molecular weight distribution of identified proteins spanned a wide range, from low-mass proteins to high-molecular-weight components, demonstrating the depth of our proteomic coverage (Figure S1F). Inter-sample correlation analyses showed strong reproducibility within experimental groups, with correlation coefficients ranging from 0.85 to 0.95 among biological replicates (Figure S1G). Principal component analysis (PCA) revealed distinct clustering patterns among the three patient groups, with the first two principal components explaining approximately 30% of total variance (Figure S1H).

**Table S1** Isolation list for PRM standard mode.

| Compound | Mass [m/z] | Formula [M] | Species | CS [z] | Polarity | Start [min] | End [min] | NCE |
| --- | --- | --- | --- | --- | --- | --- | --- | --- |
| AFVVDMMER (light) | 549.259544 |  |  | 2 | Positive |  |  | 27 |
| AHLLSLVDVMQR (light) | 691.384654 |  |  | 2 | Positive |  |  | 27 |
| IEDLPTMVTLGNSFLHK (light) | 639.004638 |  |  | 3 | Positive |  |  | 27 |
| VFAILENK (light) | 467.273835 |  |  | 2 | Positive |  |  | 27 |
| SMEIPGLR (light) | 451.741845 |  |  | 2 | Positive |  |  | 27 |
| ENFYQNWK (light) | 564.75908 |  |  | 2 | Positive |  |  | 27 |
| GDSVVYGLR (light) | 483.256174 |  |  | 2 | Positive |  |  | 27 |
| ANDESNEHSDVIDSQELSK (light) | 706.313693 |  |  | 3 | Positive |  |  | 27 |
| EFHSHEFHSHEDMLVVDPK (light) | 774.019898 |  |  | 3 | Positive |  |  | 27 |
| DSHSLTTNIMEILR (light) | 815.416879 |  |  | 2 | Positive |  |  | 27 |
| DSHSLTTNIMEILR (light) | 543.947011 |  |  | 3 | Positive |  |  | 27 |
| ALTDMPQMR (light) | 531.757169 |  |  | 2 | Positive |  |  | 27 |
| ESSSHHPGIAEFPSR (light) | 546.593737 |  |  | 3 | Positive |  |  | 27 |
| MGPTELLIEMEDWK (light) | 846.404591 |  |  | 2 | Positive |  |  | 27 |
| TMTIHNGMFFSTYDR (light) | 607.608001 |  |  | 3 | Positive |  |  | 27 |
| HGTDDGVVWMNWK (light) | 772.851175 |  |  | 2 | Positive |  |  | 27 |
| YEASILTHDSSIR (light) | 746.375537 |  |  | 2 | Positive |  |  | 27 |
| YEASILTHDSSIR (light) | 497.91945 |  |  | 3 | Positive |  |  | 27 |
| IHLISTQSAIPYALR (light) | 841.983046 |  |  | 2 | Positive |  |  | 27 |
| IHLISTQSAIPYALR (light) | 561.65779 |  |  | 3 | Positive |  |  | 27 |
| ASGYTFTSYDINWVR (light) | 890.420476 |  |  | 2 | Positive |  |  | 27 |
| SEDTAVYYC[+57.021464]AR (light) | 667.787709 |  |  | 2 | Positive |  |  | 27 |
| FGIAVLGYLNR (light) | 611.850773 |  |  | 2 | Positive |  |  | 27 |
| SPNQNVQQAAAGALR (light) | 762.897502 |  |  | 2 | Positive |  |  | 27 |
| LDAEVPTR (light) | 450.742899 |  |  | 2 | Positive |  |  | 27 |
| HLDSVLQQLQTEVYR (light) | 610.323247 |  |  | 3 | Positive |  |  | 27 |
| GAQTLYVPNC[+57.021464]DHR (light) | 765.859531 |  |  | 2 | Positive |  |  | 27 |
| VLVTGATGLLGR (light) | 578.856055 |  |  | 2 | Positive |  |  | 27 |
| AVLENNLGAAVLR (light) | 670.38825 |  |  | 2 | Positive |  |  | 27 |
| EPC[+57.021464]VESLVSQYFQTVTDYGK (light) | 1175.549016 |  |  | 2 | Positive |  |  | 27 |
| SPELQAEAK (light) | 486.753464 |  |  | 2 | Positive |  |  | 27 |
| YYTLEEIQK (light) | 593.803154 |  |  | 2 | Positive |  |  | 27 |
| FLEEHPGGEEVLR (light) | 756.37808 |  |  | 2 | Positive |  |  | 27 |
| TFIIGELHPDDRPK (light) | 546.626378 |  |  | 3 | Positive |  |  | 27 |

Notes: Compound, peptide identifier or sequence; Mass [m/z], PRM targeted precursors' mass-to-charge ratio; Formula [M], molecular formula of the neutral compound; Species, biological species or peptide origin; CS [z], charge state of the precursor ion; Polarity: ionization polarity; Start [min]: start time (in minutes) of the retention time window for precursors isolation. Empty values indicate not specified. End [min], End time (in minutes) of the retention time window. Empty values indicate not specified. NCE, normalized collision energy (in arbitrary units) used for fragmentation.

**Table S2** Descriptive characteristics of validation cohort.

| **Characteristic** | **Total**  **N = 45** | **Con**  **n = 15** | **CSKP**  **n = 15** | **CRKP**  **n = 15** |
| --- | --- | --- | --- | --- |
| Age (yrs, mean ± SD) | 53.31 ± 15.70 | 44.60 ± 13.33 | 59.40 ± 13.57 | 55.93 ± 16.87 |
| Sex (n, %) |  |  |  |  |
| Male | 35 (77.78) | 11 (73.33) | 11 (73.33) | 13 (86.67) |
| Female | 10 (22.22) | 4 (26.67) | 4 (26.67) | 2 (13.33) |
| Weight (kg, mean ± SD) | 64.62 ± 15.86 | 72.33 ± 17.23 | 56.87 ± 11.26 | 64.67 ± 15.44 |
| TBIL (μmol/L, median [IQR]) | 15.50 (11.80, 19.60) | 13.40 (11.15, 16.80) | 16.40 (14.15, 20.80) | 14.90 (10.20, 21.10) |
| ALT (U/L, median [IQR]) | 25.80 (14.00, 46.00) | 28.00 (13.00, 82.00) | 39.00 (19.50, 41.50) | 21.00 (11.00, 37.00) |
| AST (U/L, median [IQR]) | 11.00 (7.00, 20.00) | 9.40 ± 3.74 | 8.00 (7.00, 14.00) | 20.00 (15.00, 31.00) |
| Creatinine (μmol/L, median [IQR]) | 68.00 (57.00, 90.00) | 69.33 ± 14.21 | 73.47 ± 23.56 | 68.00 (43.50, 219.00) |
| BUN (mmol/L, median [IQR]) | 4.65 (3.90, 8.00) | 4.59 ± 1.97 | 4.40 (3.95, 5.30) | 10.65 (6.10, 28.70) |
| NLR (median [IQR]) | 3.09 (2.01, 6.60) | 2.11 ± 1.05 | 3.36 (2.24, 6.25) | 9.01 (4.64, 23.84) |
| PCT (ng/mL, median [IQR]) | 0.36 (0.07, 1.69) | 0.07 (0.05, 0.17) | 0.17 (0.05, 1.33) | 3.14 (1.04, 19.62) |
| CRP (mg/L, median [IQR]) | 27.50 (5.00, 98.25) | 5.00 (3.50, 12.00) | 21.00 (6.50, 55.15) | 115.74 ± 71.74 |
| WBC count (median [IQR]) | 6.59 (4.85, 8.12) | 5.95 ± 1.32 | 6.57 (3.40, 7.73) | 11.02 ± 6.76 |
| RBC count (mean ± SD) | 3.62 ± 1.21 | 4.43 ± 0.81 | 3.87 ± 1.16 | 2.55 ± 0.76 |
| Hb (median [IQR]) | 113.00 (83.00, 135.00) | 135.00 (123.00, 145.00) | 112.07 ± 29.61 | 81.53 ± 21.88 |
| RDW (median [IQR]) | 13.50 (12.70, 15.50) | 13.30 (12.30, 13.70) | 13.40 (12.85, 14.05) | 14.76 ± 1.72 |
| PLT (median [IQR]) | 220.00 (166.00, 256.00) | 247.00 (219.00, 285.50) | 239.00 (160.50, 247.50) | 166.60 ± 111.05 |
| Diabetes (n, %) |  |  |  |  |
| 0 | 32 (71.11) | 8 (53.33) | 10 (66.67) | 14 (93.33) |
| 1 | 13 (28.89) | 7 (46.67) | 5 (33.33) | 1 (6.67) |
| Heart disease (n, %) |  |  |  |  |
| 0 | 42 (93.33) | 14 (93.33) | 13 (86.67) | 15 (100.00) |
| 1 | 3 (6.67) | 1 (6.67) | 2 (13.33) | 0 (0) |
| COPD (n, %) |  |  |  |  |
| 0 | 15 (100.00) | 15 (100.00) | 15 (100.00) | 15 (100.00) |
| 1 | 0 (0) | 0 (0) | 0 (0) | 0 (0) |
| Liver disease (n, %) |  |  |  |  |
| 0 | 37 (82.22) | 9 (60.00) | 15 (100.00) | 13 (86.67) |
| 1 | 8 (17.78) | 6 (40.00) | 0 (0) | 2 (13.33) |
| Cerebrovascular disease (n, %) |  |  |  |  |
| 0 | 34 (75.56) | 7 (46.67) | 15 (100.00) | 12 (80.00) |
| 1 | 11 (24.44) | 8 (53.33) | 0 (0) | 3 (20.00) |
| Kidney disease (n, %) |  |  |  |  |
| 0 | 43 (95.56) | 14 (93.33) | 15 (100.00) | 14 (93.33) |
| 1 | 2 (4.44) | 1 (6.67) | 0 (0) | 1 (6.67) |

Abbreviations: Con, culture-negative controls; CSKP, carbapenem-susceptible Klebsiella pneumoniae; CRKP, carbapenem-resistant Klebsiella pneumoniae; TBIL, total bilirubin; ALT, Alanine aminotransferase; AST, Aspartate aminotransferase; Creatinine; BUN, blood urea nitrogen; NLR, neutrophil-to-lymphocyte ratio; PCT, procalcitonin; CRP, C-reactive protein; WBC count, white blood cell count; RBC count, red blood cell count; Hb, hemoglobin; RDW, red cell distribution width; PLT, platelet count; COPD, chronic obstructive pulmonary disease.

**Table S3** Performance of the candidate protein panel and clinical markers in distinguishing sepsis groups.

| **Variables** | **CSKP vs Con**  **(AUC, 95% CI)** | **CRKP vs Con**  **(AUC, 95% CI)** | **CRKP vs CSKP**  **(AUC, 95% CI)** |
| --- | --- | --- | --- |
| Protein panel | 0.920 (0.736–1.000) | 0.840 (0.568–1.000) | 0.920 (0.736–1.000) |
| PCT | 0.840 (0.568–1.000) | 0.940 (0.813–1.000) | 0.640 (0.197–1.000) |
| PCT+Protein panel | 0.840 (0.568-1.000) | 1.000 (1.000–1.000) | 0.920 (0.736–1.000) |
| CRP | 0.840 (0.517–1.000) | 1.000 (1.000–1.000) | 0.560 (0.100–1.000) |
| CRP+Protein panel | 0.880 (0.658–1.000) | 1.000 (1.000–1.000) | 0.640 (0.329–0.951) |
| NLR | 0.920 (0.736-1.000) | 0.960 (0.868–1.000) | 0.520 (0.105–0.935) |
| NLR+Protein panel | 0.700 (0.303–1.000) | 1.000 (1.000–1.000) | 0.640 (0.278–1.000) |
| PCT+CRP+NLR | 0.920 (0.736–1.000) | 1.000 (1.000–1.000) | 0.520 (0.070–0.970) |
| PCT+CRP+NLR+protein panel | 0.920 (0.736–1.000) | 1.000 (1.000–1.000) | 0.680 (0.330–1.000) |

Abbreviations: Con, culture-negative controls; CSKP, carbapenem-susceptible Klebsiella pneumoniae; CRKP, carbapenem-resistant Klebsiella pneumoniae; NLR, neutrophil-to-lymphocyte ratio; PCT, procalcitonin; CRP, C-reactive protein; AUC, area under the curve; CI, confidence interval.

**Table S4** Targeted PRM quantification and differential abundance analysis of model-derived proteins in the independent validation cohort.

| **Gene** | CSKP vs Con | |  | CRKP vs Con | |  | CRKP vs CSKP | |
| --- | --- | --- | --- | --- | --- | --- | --- | --- |
|  | Log2 FC | P value |  | Log2 FC | P value |  | Log2 FC | P value |
| CYB5A | 0.08 | 0.78 |  | 0.47 | 0.10 |  | 0.39 | 0.20 |
| APOA2 | -3.69 | **0.02** |  | -2.94 | 0.08 |  | 0.74 | 0.59 |
| IGHV1-8 | -0.03 | 0.96 |  | 0.49 | 0.35 |  | 0.52 | 0.30 |
| ITGA2 | -2.31 | 0.21 |  | 0.25 | 0.57 |  | 2.56 | 0.16 |
| IGFBP6 | -5.13 | 0.12 |  | 4.87 | **0.03** |  | 10.00 | **<0.01** |

**Figure S1** Population Characteristics and Data Overview.

(A) Quantitative distribution of protein quantity.

(B) Quantitative distribution of peptide quantity.

(C) Peptide length distribution.

(D) Peptide number distribution.

(E) Missing cleavages distribution.

(F) Protein mass distribution.

(G) Heatmap of correlation between samples.

(H) Principal component analysis of quantitative proteins.


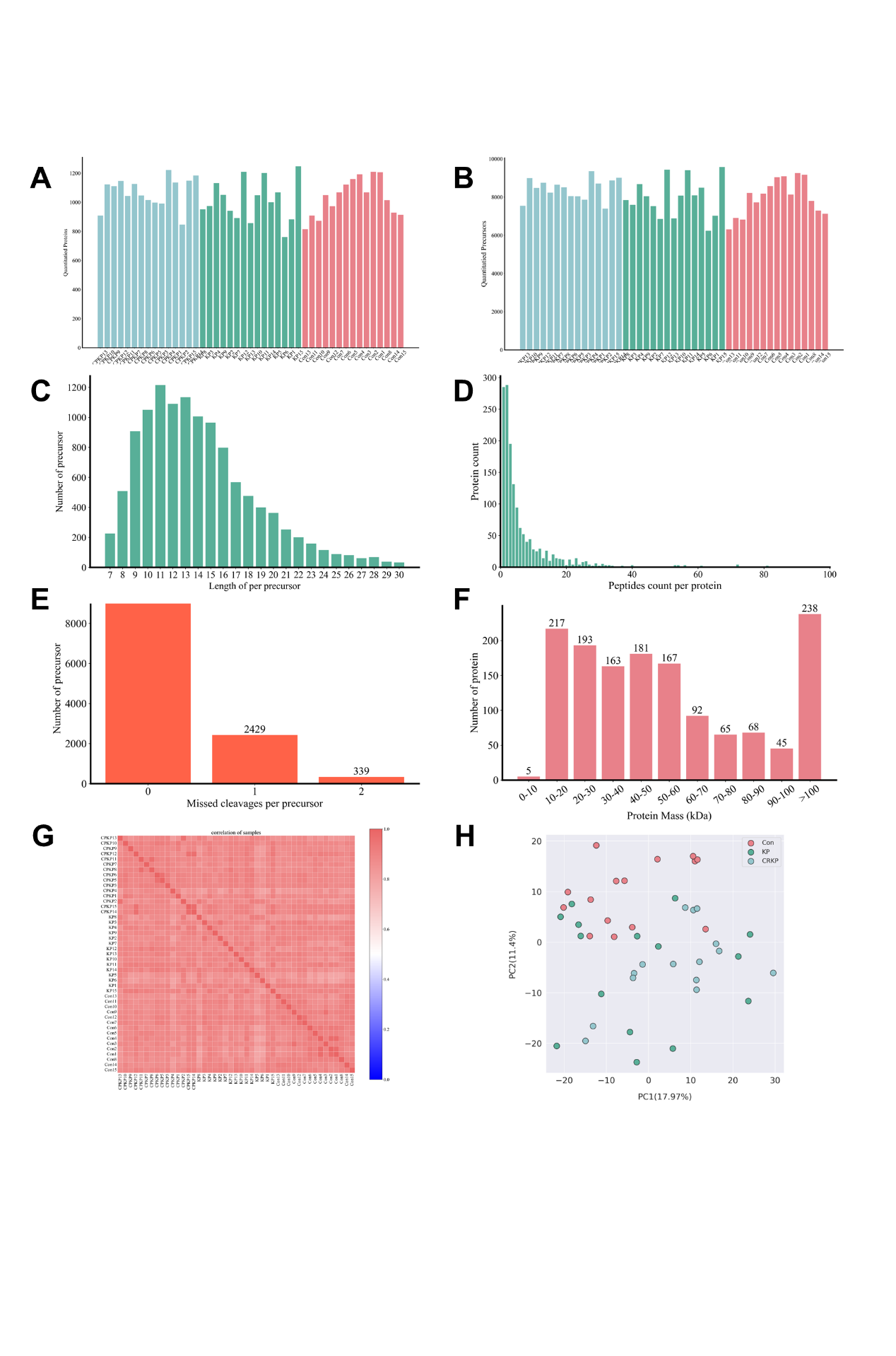


**Figure S2** Calibration curves and decision curves for the final models in the test set.

1. Calibration curve of the test set for the CSKP group and the Con group.
2. Decision curve of the test set for the CSKP group and the Con group.
3. Calibration curve of the test set for the CRKP group and the Con group.
4. Decision curve of the test set for the CRKP group and the Con group.
5. Calibration curve of the test set for the CRKP group and the CSKP group.
6. Decision curve of the test set for the CRKP group and the CSKP group.

**
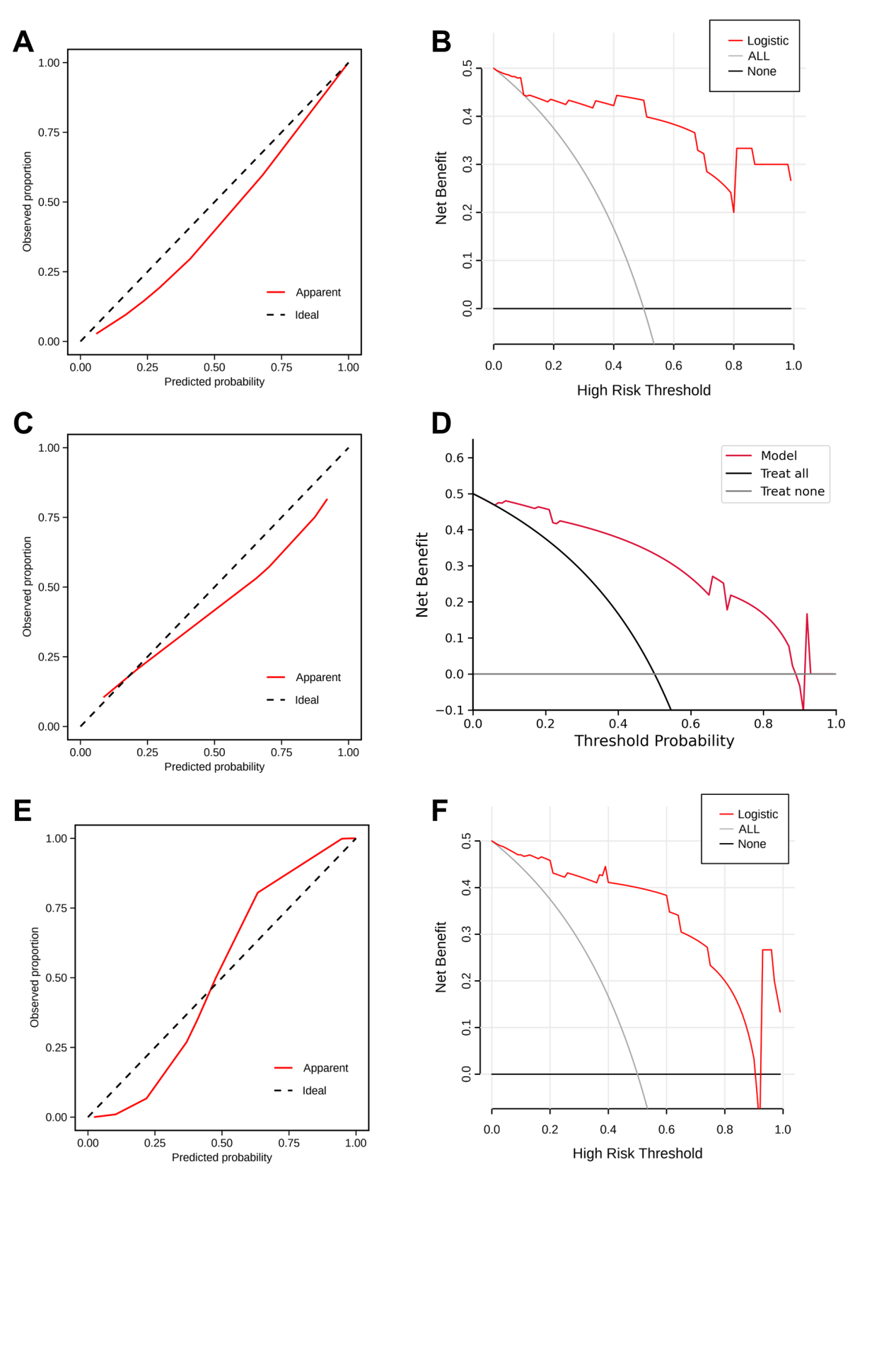
**

**Figure S3** Validation of APOA2 and IGFBP6 expression patterns and diagnostic performance in the validation cohort.

(A) Box plots showing the relative expression levels of APOA2 across the Con, CSKP, and CRKP groups. *: P < 0.05; **: P < 0.01; ***: P < 0.001.

(B) Box plots showing the relative expression levels of IGFBP6 across the Con, CSKP, and CRKP groups. *: P < 0.05; **: P < 0.01; ***: P < 0.001.

(C) ROC curve evaluating the diagnostic performance of APOA2 for discriminating between CSKP and Con group.

(D) ROC curve evaluating the diagnostic performance of IGFBP6 for discriminating between CRKP and CSKP group.


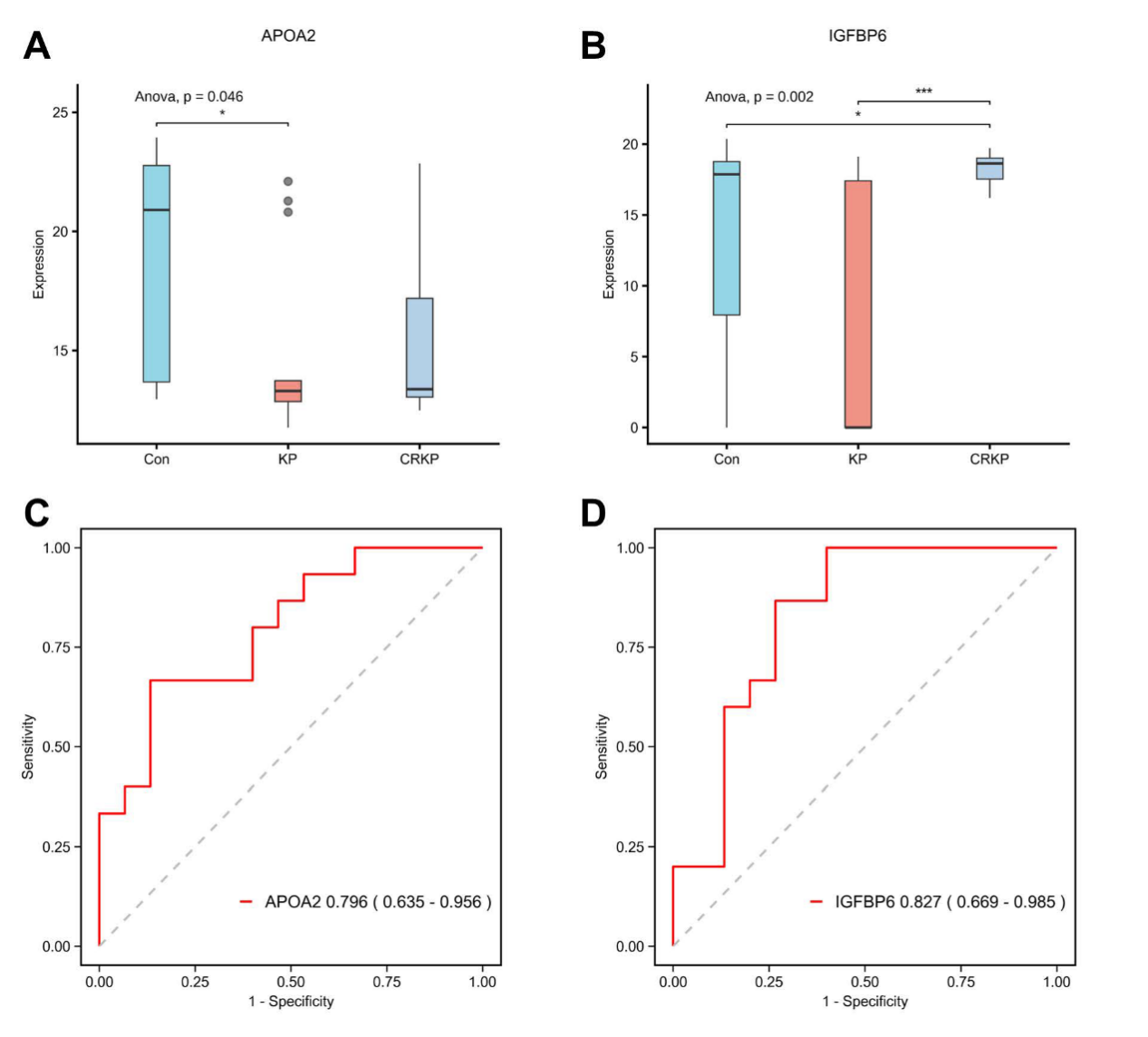


**Figure S4** Drug-protein interaction network of trend proteins.

(A) Interaction network diagram of drug-targeted proteins. Purple dots were drugs, red dots were trend proteins, and gray solid lines indicated protein-drug interactions or protein-drug interactions.

(B) Interaction network diagram of Top5 multi-target drugs.

(C) Interaction network diagram of disease targets and drugs.

(D) Interaction network of ion channel drug targets with drugs.

(E) Interaction network of receptor drug targets with drugs.


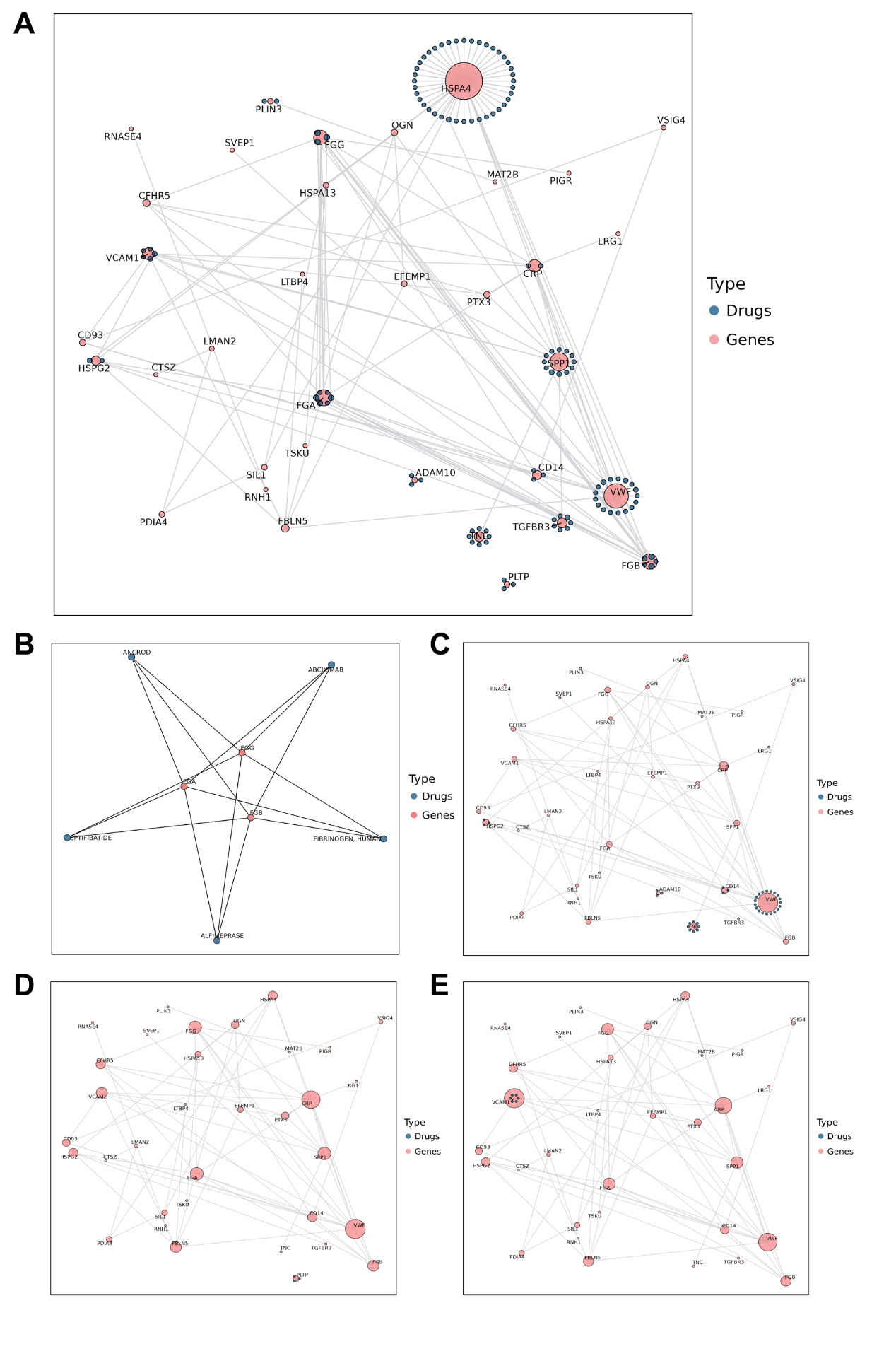


**Figure S5** Enrichment analysis and cluster analysis of DEMs.

(A) KEGG pathway enrichment analysis for DEMs between KP and Con group. The top 20 pathways ranked by P-value (from smallest to largest) were selected for display. The horizontal axis represents the Rich Factor corresponding to each pathway, and the vertical axis shows the names of the pathways (sorted by P-value). The color of the dots reflects the magnitude of the P-value: the redder the dot, the more significant the enrichment. The size of the dots represents the number of DEMs enriched in the pathway.

(B) KEGG pathway enrichment analysis for DEMs between CRKP and Con group.

(C) K-means trend cluster plot of DEMs. The horizontal axis represents sample groups, the vertical axis represents the relative content of metabolites, and "Sub class" denotes the category number of metabolites with the same variation trend.


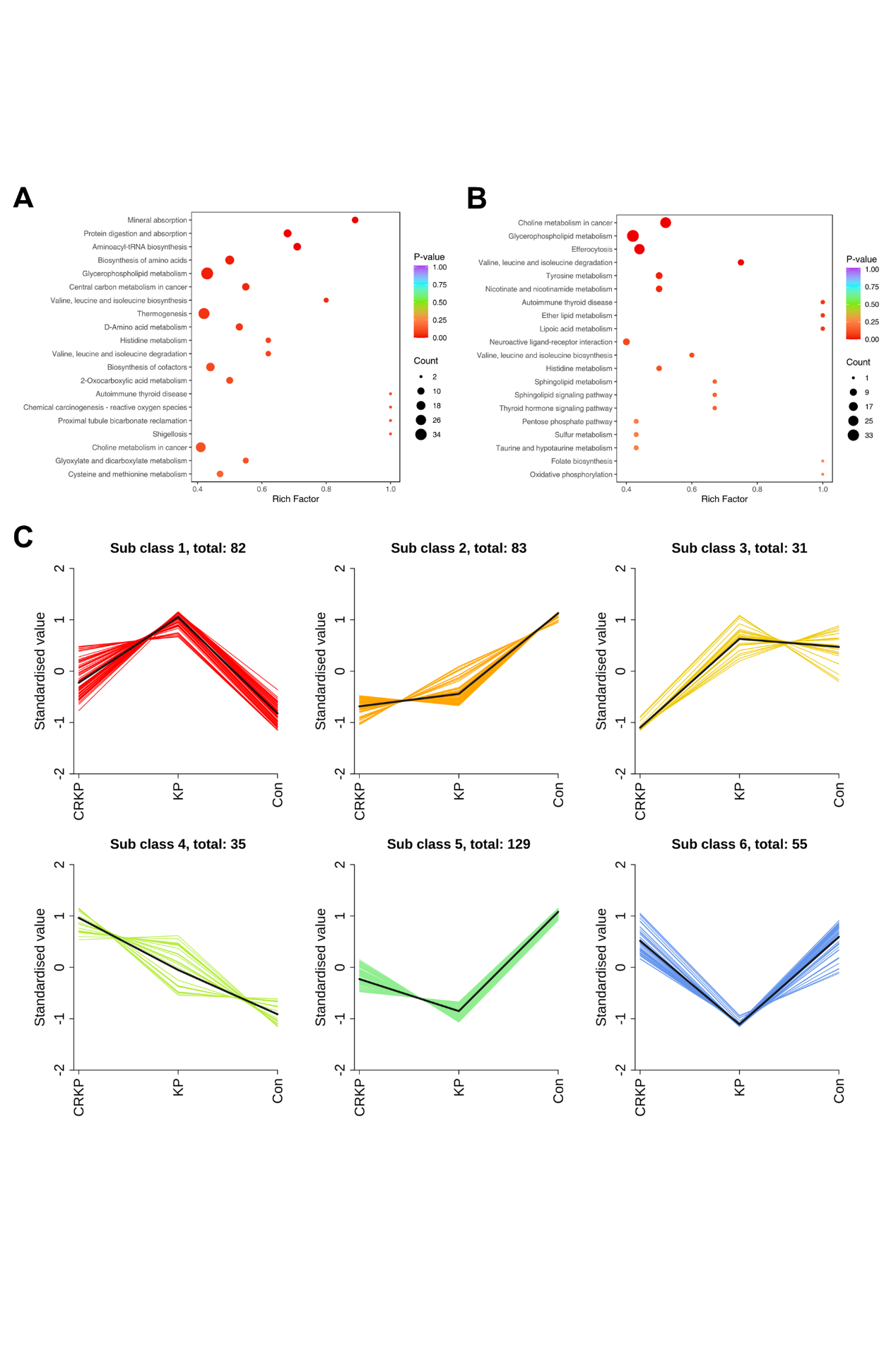


**Figure S6** Schematic diagram of the One carbon pool by folate pathway (KEGG Accession: map00670).


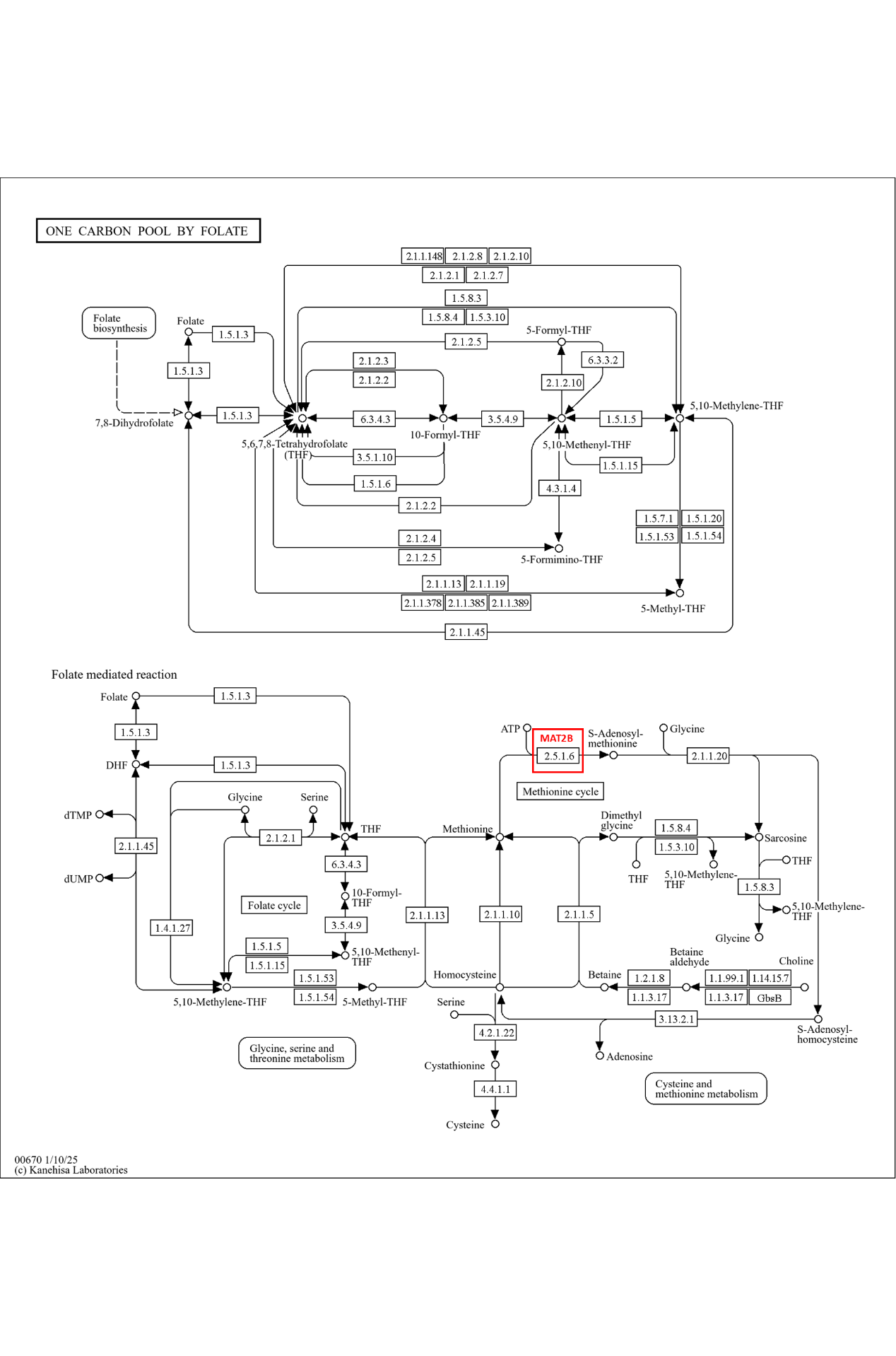


**Figure S7** Schematic diagram of the Cysteine and methionine metabolism pathway (KEGG Accession: map00270).


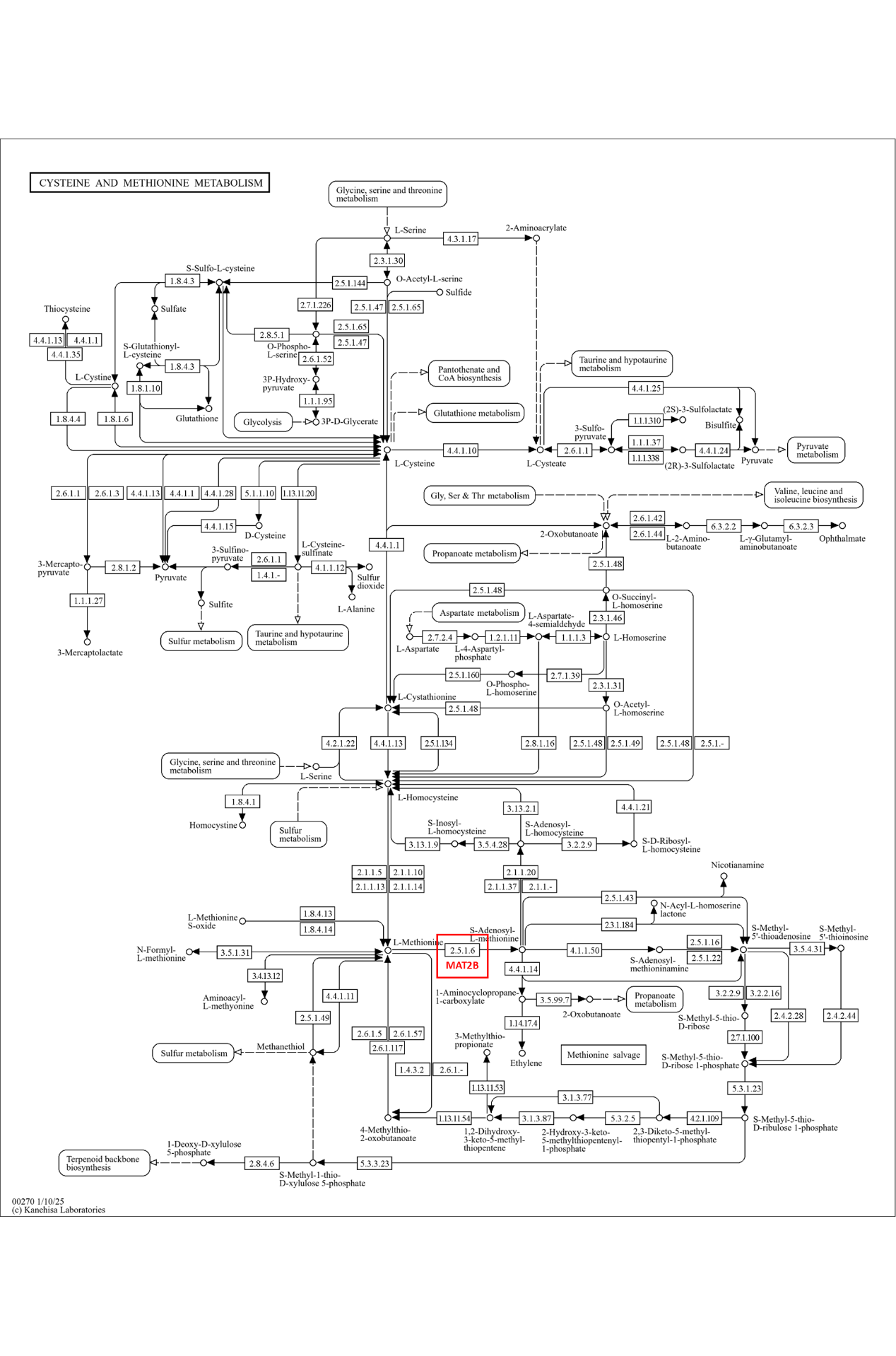


**Figure S8** KEGG pathway integrated analysis of proteomics and metabolomics.

(A) Venn diagram of the number of common pathways between metabolites (blue) and proteins (pink).

(B) Combined KEGG pathway analysis results of metabolites and proteins with gradually increasing expression levels. The relative quantities of proteins and metabolites in the common pathways were shown within the circles.

(C) Box plot of MAT2B expression levels in different groups. ns: No significant difference; *: P < 0.05; **: P < 0.01; ***: P < 0.001.


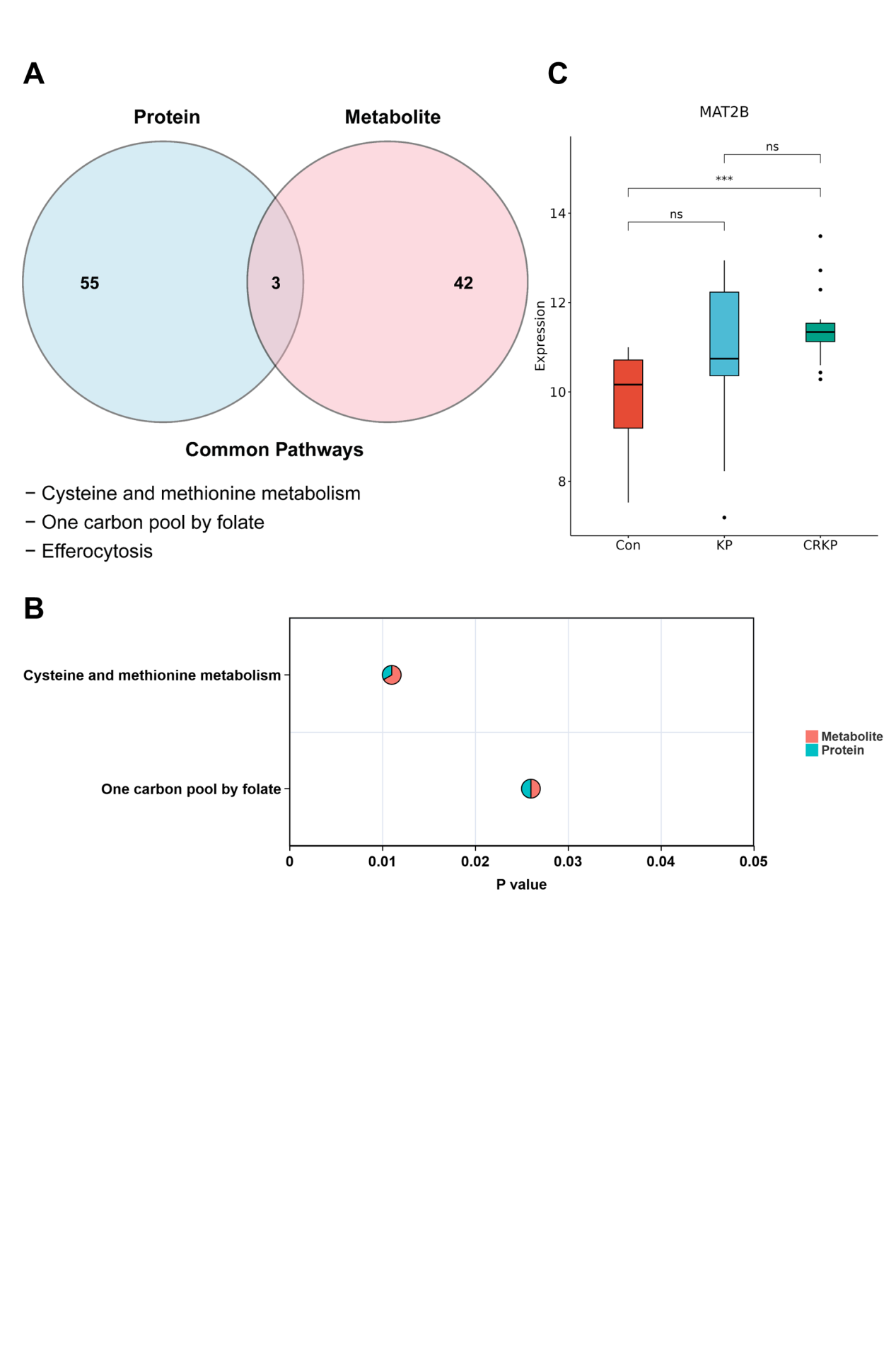

Supplement: Supplementary file 2 [file Table1.docx]
